# The Global Acetylome of the Human Pathogen *Vibrio cholerae* V52 Reveals Lysine Acetylation of Major Transcriptional Regulators

**DOI:** 10.3389/fcimb.2017.00537

**Published:** 2018-01-11

**Authors:** Carsten Jers, Vaishnavi Ravikumar, Mateusz Lezyk, Abida Sultan, Åsa Sjöling, Sun N. Wai, Ivan Mijakovic

**Affiliations:** ^1^Department of Biotechnology and Biomedicine, Technical University of Denmark, Kongens Lyngby, Denmark; ^2^Novo Nordisk Foundation Center for Biosustainability, Technical University of Denmark, Kongens Lyngby, Denmark; ^3^Department of Chemical and Biochemical Engineering, Technical University of Denmark, Kongens Lyngby, Denmark; ^4^Department of Microbiology, Tumor and Cell Biology, Karolinska Institutet, Stockholm, Sweden; ^5^Department of Molecular Biology, Umeå University, Umeå, Sweden; ^6^Systems and Synthetic Biology Division, Department of Biology and Biological Engineering, Chalmers University of Technology, Gothenburg, Sweden

**Keywords:** *Vibrio cholerae*, pathogen, bacteria, lysine acetylation, acetylome, mass spectrometry, proteomics, virulence

## Abstract

Protein lysine acetylation is recognized as an important reversible post translational modification in all domains of life. While its primary roles appear to reside in metabolic processes, lysine acetylation has also been implicated in regulating pathogenesis in bacteria. Several global lysine acetylome analyses have been carried out in various bacteria, but thus far there have been no reports of lysine acetylation taking place in the important human pathogen *Vibrio cholerae*. In this study, we analyzed the lysine acetylproteome of the human pathogen *V. cholerae* V52. By applying a combination of immuno-enrichment of acetylated peptides and high resolution mass spectrometry, we identified 3,402 acetylation sites on 1,240 proteins. Of the acetylated proteins, more than half were acetylated on two or more sites. As reported for other bacteria, we observed that many of the acetylated proteins were involved in metabolic and cellular processes and there was an over-representation of acetylated proteins involved in protein synthesis. Of interest, we demonstrated that many global transcription factors such as CRP, H-NS, IHF, Lrp and RpoN as well as transcription factors AphB, TcpP, and PhoB involved in direct regulation of virulence in *V. cholerae* were acetylated. In conclusion, this is the first global protein lysine acetylome analysis of *V. cholerae* and should constitute a valuable resource for in-depth studies of the impact of lysine acetylation in pathogenesis and other cellular processes.

## Introduction

Protein acetylation is an abundant post translational modification. In bacteria, protein acetylation can be achieved by two distinct mechanisms. One is an enzymatic process, catalyzed by a protein acetyltransferase, where the acetyl group of acetyl coenzyme A is transferred to a lysine residue of a target protein. The other is non-enzymatic, where the acetyl group of acetyl phosphate is transferred directly to the lysine residue. In both cases, protein acetylation can be reversed by the action of a protein deacetylase.

There is an accumulating number of examples of characterized acetylation events affecting essentially all parts of the bacterial cell and it is thus clear that acetylation is an important regulatory modification in bacteria. Mass spectrometry-based analysis has demonstrated that a very large subset of bacterial proteins can be acetylated as exemplified by the work on *Mycoplasma pneumoniae* where ~32% of proteins are acetylated (van Noort et al., 2012). In *Escherichia coli*, for many identified sites only a low fraction of the protein molecules are acetylated and many sites are not targeted by deacetylases (Weinert et al., 2013; Meyer et al., 2016). At present, it is thus not clear whether all the sites identified in global acetylome studies are in fact regulatory (Hentchel and Escalante-Semerena, 2015).

There is an increasing body of evidence implicating protein acetylation in bacterial pathogenesis. Mass spectrometry-based acetylation studies have demonstrated that many virulence factors of these organisms are acetylated (Ren et al., 2017). In *Mycobacterium tuberculosis*, lysine acetylation is involved in regulation of cell wall fatty acids synthesis, which in turn is implicated in pathogenicity (Liu et al., 2014). Additionally, mutation of a lysine deacetylase (MRA_1161) leads to a defect in biofilm formation by *M. tuberculosis* (Liu et al., 2014). In *Salmonella typhimurium* the transcriptional regulator HilD is acetylated by the acetyltransferase Pat which increases protein stability but reduces DNA binding activity. During infection, the level of HilD acetylation decreases which leads to increased virulence of *S. typhimurium* (Sang et al., 2017). In the same bacterium, the two-component system response regulator PhoP is also acetylated and as for HilD, acetylation decreases its DNA binding activity (Ren et al., 2016). Upon phagocytosis by macrophages, PhoP acetylation decreases and this is critical for survival in the host (Ren et al., 2016). In *E. coli*, the transcription factor RcsB that controls colanic acid capsule synthesis is acetylated on lysine which leads to reduced DNA binding activity (Thao et al., 2010). *Porphyromonas gingivalis* is an important causal agent of periodontal disease. *P. gingivalis* VimA is an important regulator that modulates several processes pertaining to virulence. It has been suggested that the multifunctionality of the protein could be facilitated by protein acetylation (Aruni et al., 2013).

*Vibrio cholerae* is the causative agent of the diarrheal disease cholera that annually leads to an estimated 3 million cases and 100,000 deaths (Ali et al., 2015). Regulation of virulence is well-studied in *V. cholerae* and appears to a large extent to take place at the transcriptional level via a regulatory cascade leading to activation of ToxT that regulates expression of the important virulence genes encoding cholera toxin and toxin co-regulated pilus (Silva and Benitez, 2016). Expression of *toxT* is enhanced by TcpP/H that is in turn under transcriptional control of AphAB. Other transcriptional regulators H-NS, HapR, CRP, Lrp, and PhoB modulate virulence gene transcription in response to various conditions (Rutherford and Bassler, 2012; Almagro-Moreno et al., 2015; Silva and Benitez, 2016). While regulation by post translational modifications, notably His/Asp phosphorylation, has been reported, to our knowledge, there have been no reports of regulation mediated by protein acetylation in *V. cholerae*. Recently, *Vibrio parahemolyticus* was subjected to an analysis of protein acetylation that identified 1,413 lysine acetylation sites in 656 proteins (Pan et al., 2014) indicating that protein acetylation could also be an important regulatory post translational modification in the related pathogen *V. cholerae*.

In this study, we wanted to address the hypothesis that protein acetylation is an important regulatory post translational modification in *V. cholerae*. To do so, we chose the clinical isolate *V. cholerae* V52 that was responsible for an outbreak of cholera-like diarrheal illness in Sudan, with 460 cases leading to 125 deaths (Zinnaka and Carpenter, 1972). Studies have mainly focused on the epidemic *V. cholerae* strains belonging to serogroups O1 and O139. While non-O1/non-O139 *V. cholerae* rarely cause outbreaks, they represent an emerging threat and are of increasing concern in both endemic and non-endemic areas. Identification of virulence gene modulation in the non-O1/non-O139 serogroups of *V. cholerae* is very important since these isolates with epidemic potential may emerge in the future, as seen in the case of the O139 serogroup.

By mass spectrometry analysis, we identified 3,402 acetylation sites on 1,240 proteins. Our bioinformatics analysis indicated that several of these acetylation sites could serve a regulatory function. This study thus provides evidence that protein acetylation is an important post translational modification in *V. cholerae* and provides a foundation for further in-depth studies of the functional roles of protein acetylation in virulence and other cellular processes.

## Materials and methods

### Strain and growth condition

In this study, we used the pathogenic strain *V. cholerae* V52 that was isolated in a cholera outbreak in Sudan in 1968 (Zinnaka and Carpenter, 1972). The strain was a kind gift from Dr. Jun Zhu, Pennsylvania University, US. An overnight culture, grown in LB medium at 37°C with shaking at 180 rpm, was used as a pre-inoculum for the main culture, which was grown under same conditions. For the western blot analysis, the cultures were harvested at five different time points, namely T1 (OD_600_ of 0.1), T2 (OD_600_ of 0.5), T3 (OD_600_ of 1.0), T4 (OD_600_ of 1.3), and T5 (OD_600_ of 1.5). The experiment was performed three times, and a representative experiment is shown. For the proteomic analysis, the cultures were grown until mid-logarithmic phase (OD_600_ of 0.5) and stationary phase (OD_600_ of 1.2). For this experiment two biological replicates were performed.

### Protein extraction

Harvested cells were spun down at 5,000 rpm for 15 min. The pellet was lysed using a 4% sodium dodecyl sulfate buffer solution prepared in 100 mM triethylammonium bicarbonate (pH 8.0), containing 10 mM ethylenediaminetetraacetic acid and a protease cocktail (Roche). The cell extract was boiled for 10 min at 90°C and then briefly sonicated on ice. This was then followed by centrifugation for 30 min at 13,400 rpm at 4°C. The supernatant was treated with chloroform and methanol to obtain a clean protein precipitate that was dissolved in a buffer containing 6 M urea, 2 M thiourea in 10 mM tris-HCl pH 7.5. The protein concentration was determined using the Bradford protein assay (Biorad), using bovine serum albumin as a standard.

### Western blot

For each sample point, 15 μg of extracted protein was separated on an SDS-polyacrylamide gel. The proteins were transferred to a nitrocellulose membrane using the iBlot dry blotting system (Invitrogen). Then the membrane was blocked for 1 h with TBST (10 mM Tris, 150 mM NaCl, and 0.1% Tween20, pH 7.6) containing 2% skim milk powder. After washing with TBST, the membrane was incubated with anti-acetyl lysine antibody (Immunechem, cat. no. ICP0380) diluted 1:1000 in TBST with 0.1% skim milk for 2 h. The washed membrane was incubated with goat anti-rabbit antibody conjugated with horse radish peroxidase (Immunechem, cat. no. ICP9803) for 1 h. Finally, the antibody was detected using the ECL prime western blotting detection reagent (GE Healthcare).

### Protein digestion and immuno-enrichment

For each sample, 7.5 mg protein was reduced with 1 mM dithiothreitol for 1 h at room temperature and then alkylated with 5.5 mM iodoacetamide for 1 h at room temperature, in the dark. Proteins were then digested with Lys-C (Wako) (1:100 w/w) for 3 h at room temperature. The samples were diluted with four volumes of 20 mM ammoniumbicarbonate and digested overnight with trypsin (Promega) (1:100 w/w). After digestion, the sample was acidified to pH 2.7 with triflouroacetic acid, incubated at room temperature for 10 min and centrifuged (2,500 g, 5 min) to remove any precipitate. The resulting peptide solution was loaded on a C18 Sep-Pak column equilibrated with 2% acetonitrile and 1% trifluouracetic acid in water. The bound peptides were washed with 0.5% acetic acid, eluted with 80% acetonitrile and evaporated to a final volume of ~400 μL by vacuum centrifugation.

For lysine acetylation enrichment, we used an anti-acetyl lysine antibody immobilized on agarose (Immunechem, cat. no. ICP0388). The 400 μL sample was mixed with 10x IP buffer (500 mM MOPS (pH 7.2), 100 mM sodium phosphate, and 500 mL NaCl) and 2480 μL water, mixed with acetyl lysine antibody agarose pre-washed with IP buffer and incubated on a rotating wheel over night at 4°C. On a spin column, the agarose was washed three times with IP buffer, and three times with water. For elution, the agarose-bound peptides were incubated twice for 10 min each at room temperature with 40 μL of 0.15% trifluoroacetic acid.

### Mass spectrometry

Mass spectrometry analysis was performed by Carina Sihlbom at the Proteomics Core Facility, Sahlgrenska Academy, University of Gothenburg, Gothenburg, Sweden. Samples were analyzed on an Elite mass spectrometer (Thermo Fisher Scientific) interfaced with Easy nLC 1000 liquid chromatography system (Thermo Fisher Scientific). Peptides were separated using an in-house constructed C18 analytical column (300 × 0.075 mm I.D., 3 μm, Dr. Maisch, Germany) using a gradient from 4% to 28% acetonitrile in 0.2% formic acid over 45 min followed by an increase to 80% acetonitrile in 0.2% formic acid for 5 min at a flow of 300 nL/min. Precursor ion mass spectra were acquired at 120K resolution and MS/MS analysis was performed in a data-dependent mode where the 10 most intense precursor ions were selected for fragmentation using CID at a collision energy of 35. Charge states 2 to 4 were selected for fragmentation, and dynamic exclusion was set to 15 s.

### Database search

To obtain the most representative proteome for *V. cholerae* V52, a non-redundant database of 3756 proteins from two available proteomes was compiled. Proteomes UP000005193 and UP000178081 were downloaded from UniProt Proteomes database and CD-HIT with default settings was used to cluster proteins at sequence identity threshold of 1 (Fu et al., 2012). Acquired mass spectrometry spectra were processed using MaxQuant (v. 1.5.8.3). A preliminary experiment was conducted and this preliminary data was appended to our main dataset during database search to help in identifications. Database search was performed against the compiled *V. cholerae* V52 database with a reverse decoy database and 245 common contaminating proteins. Trp/P and LysC/P were specified as the protease and three missed cleavages were allowed. Carbamidomethylation on cysteines was defined as a fixed modification and methionine oxidation, N-terminal protein acetylation, and Lys acetylation were specified as variable modifications. A false discovery rate of 0.01 was applied at the peptide, protein and acetylated site level. All other parameters in the analysis were default settings.

### Bioinformatics analyses

To search for lysine protein acetyltransferases and deacetylases, we used the NCBI Conserved domains search tool (Marchler-Bauer et al., 2011). Pairwise sequence alignments were done using EMBOSS stretcher and multiple sequence alignments using CLUSTAL Omega (Sievers et al., 2011) available on the EMBL-EBI homepage (Li et al., 2015). Overrepresentation analysis of gene ontology (GO) terms was done using the PANTHER (v. 12.0) software tools (Mi et al., 2017). The *V. cholerae* V52 protein sequences were scored against the Panther HMM library of families and subfamilies using the HMM-based search tool. In total, 3,085 of 3,756 proteins (82.1%) were mapped including 1,165 of the 1,240 acetylated proteins (94.0%). Subsequently, the over-representation test was done using default settings with acetylated proteins as “analyzed list” and the *V. cholerae* V52 proteome as “reference list.” To analyse sequence motifs surrounding acetylation sites, we used the tools Motif-x (v. 1.2) (Schwartz and Gygi, 2005) and pLogo (O'Shea et al., 2013). A 21 amino acid sequence window was chosen, the compiled *V. cholerae* V52 proteome was used as background, and otherwise default settings were applied. Homology models were generated using the MPI bioinformatics toolkit (Alva et al., 2016). Protein sequences were submitted to HHpred and the five top hits were forwarded to Modeler in order to obtain the homology models. Figures of structures and homology models were made using PyMol v1.8.0.5 (Schrödinger). For AphB, VctP, and CheY, the PDB files 3SZP, 3TEF, and 3TO5 were used. The substrate bound to VctP and the interaction of CheY with CheZ (amino acids 205–215) were illustrated by superimposition with PDB files 5A5D and 2FKA, respectively. To depict CRP and IHF interaction with DNA, the homology models were superimposed with the structures 3MZH and 1IHF, respectively. And finally to identify the interaction surface of PhoB, the homology model was superimposed with structure 1ZES.

## Results

### *V. cholerae* genes encode potential protein lysine acetylases and deacetylases

Protein acetylation has previously been shown to be an abundant post translational modification in several bacteria including *V. parahemolyticus* and thus we in this study wanted to assess whether protein acetylation also takes place in *V. cholerae*. It is by now well-known that bacterial protein acetylation can take place due to either enzyme catalyzed acetylation and deacetylation by protein lysine acetylases and deacetylases as well as by chemical acetylation employing the metabolite acetyl phosphate (Weinert et al., 2013). To address whether protein acetylation could be a regulatory post translational modification in *V. cholerae* we first wanted to establish whether protein acetyltransferases and protein deacetylases are encoded in the genome of the bacterium.

With respect to protein deacetylases, we identified two enzymes namely a putative NAD^+^-dependent sirtuin-like protein deacetylase (KNH50969) and a putative NAD^+^-independent protein deacetylase (KNH50718) (Supplementary Table 1). All presently known bacterial protein acetyltransferases are characterized by the presence of the GCN5-related N-acetyltransferase (GNAT) domain and using the NCBI Domain search tool, we identified 38 proteins containing this domain (Supplementary Table 2). It is worth noting that GNAT domain proteins besides catalyzing protein acetylation can also be catalyzing acylation of other compounds such as peptides and small molecules (Favrot et al., 2016).

### Protein acetylation is detectable in *V. cholerae*

**Figure 1:**
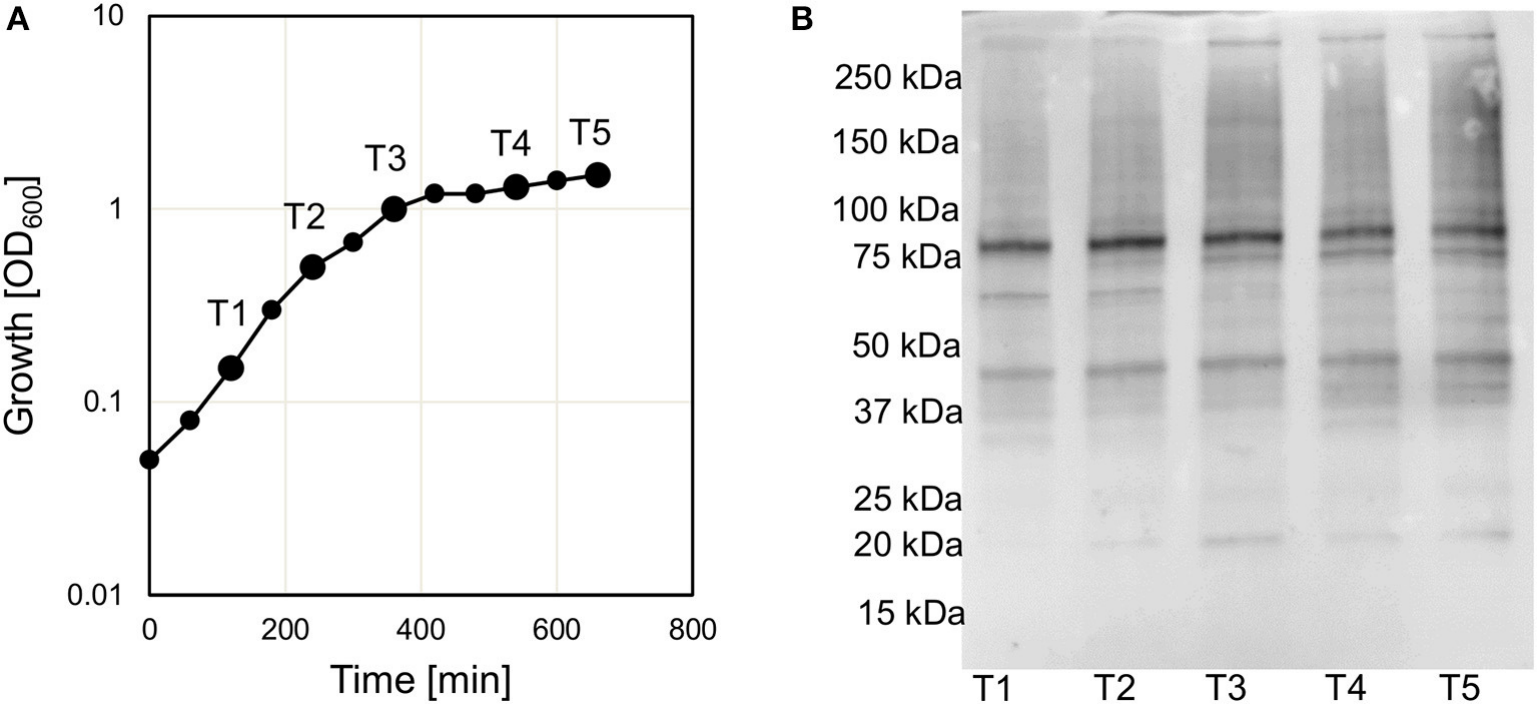
Acetylation in *V. cholerae*. **(A)** Shows the growth curve of *V. cholerae* V52 in LB medium. Sample points T1–5 are indicated on the figure. **(B)** Shows the level of acetylation at different time points (T1–T5) as analyzed by Western blot using an anti-acetyl lysine antibody. The experiment was performed three times and a representative figure is shown. A separate gel was stained to assure similar levels of protein (Supplementary Figure 1).

The fact that genes encoding protein deacetylases and potentially also protein acetyltransferases were present in *V. cholerae* would indicate a regulatory role of protein acetylation in this organism. To confirm that protein acetylation takes place in *V. cholerae* we initially analyzed the acetylation by western blot using an anti-acetyl lysine antibody. In *E. coli* it has been shown that the level of acetylation tends to increase in the stationary phase (Yu et al., 2008). We therefore cultured *V. cholerae* in LB and analyzed the acetylation levels at different stages of the growth curve including exponential, transition and stationary phase (Figure 1 and Supplementary Figure 1). Based on the western blot experiment, we could conclude that protein acetylation was detected in *V. cholerae*. When considering the different growth stages sampled, there was no pronounced difference in the level of acetylation.

### Proteomics reveals extensive protein acetylation in *V. cholerae*

**Figure 2:**
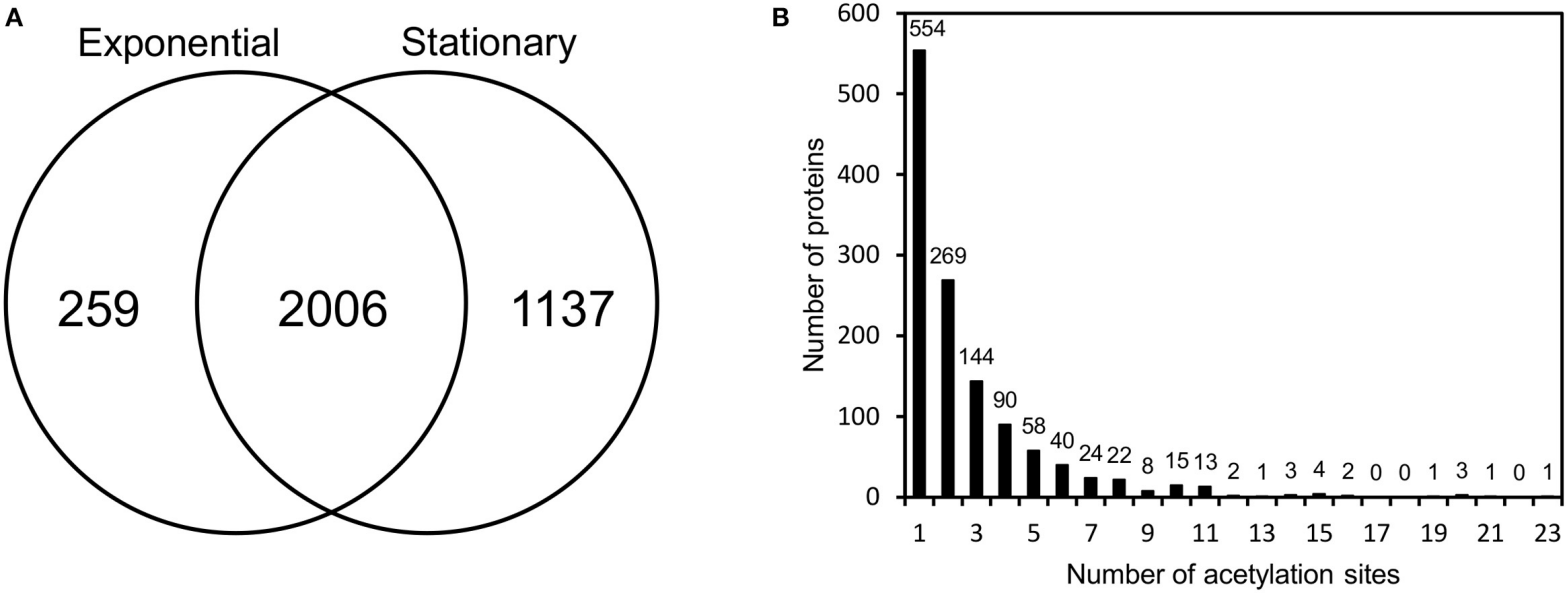
Acetylproteome of *V. cholerae* V52. **(A)** Acetylation sites identified in exponential and early stationary growth phase. **(B)** Distribution of acetylated proteins based on the number of acetylation sites.

To provide a deeper understanding of protein acetylation in *V. cholerae*, we next performed a global, site-specific analysis of the acetylproteome. To this end, we collected samples at the mid-exponential growth phase as well as in the stationary phase. These samples were digested, peptides were enriched for acetylated peptides using an anti-acetyl lysine antibody and samples were analyzed by mass spectrometry. The experiment was done in duplicates and there was a good correlation between the replicates (Pearson coefficients ranging from 0.82 to 0.91) (Supplementary Figure 2). This allowed for the detection of a total of 1,240 acetylated proteins and 3,402 acetylation sites (Figure 2A and Supplementary Table 3). A large overlap between the two conditions was observed, but it was evident that more sites were detected in the stationary phase. Of the 1,240 acetylated proteins, more than half were acetylated on two or more sites and one protein had 23 acetylation sites (Figure 2B). To address if simple protein characteristics played a role in directing acetylation, we analyzed whether there was a correlation between the number of acetylation sites and the total number of lysines in the proteins and whether secondary structure played a role. This however did not appear to be the case (Supplementary Figure 3).

### Positively charged residues are over-represented in the vicinity of acetylation sites

**Figure 3:**
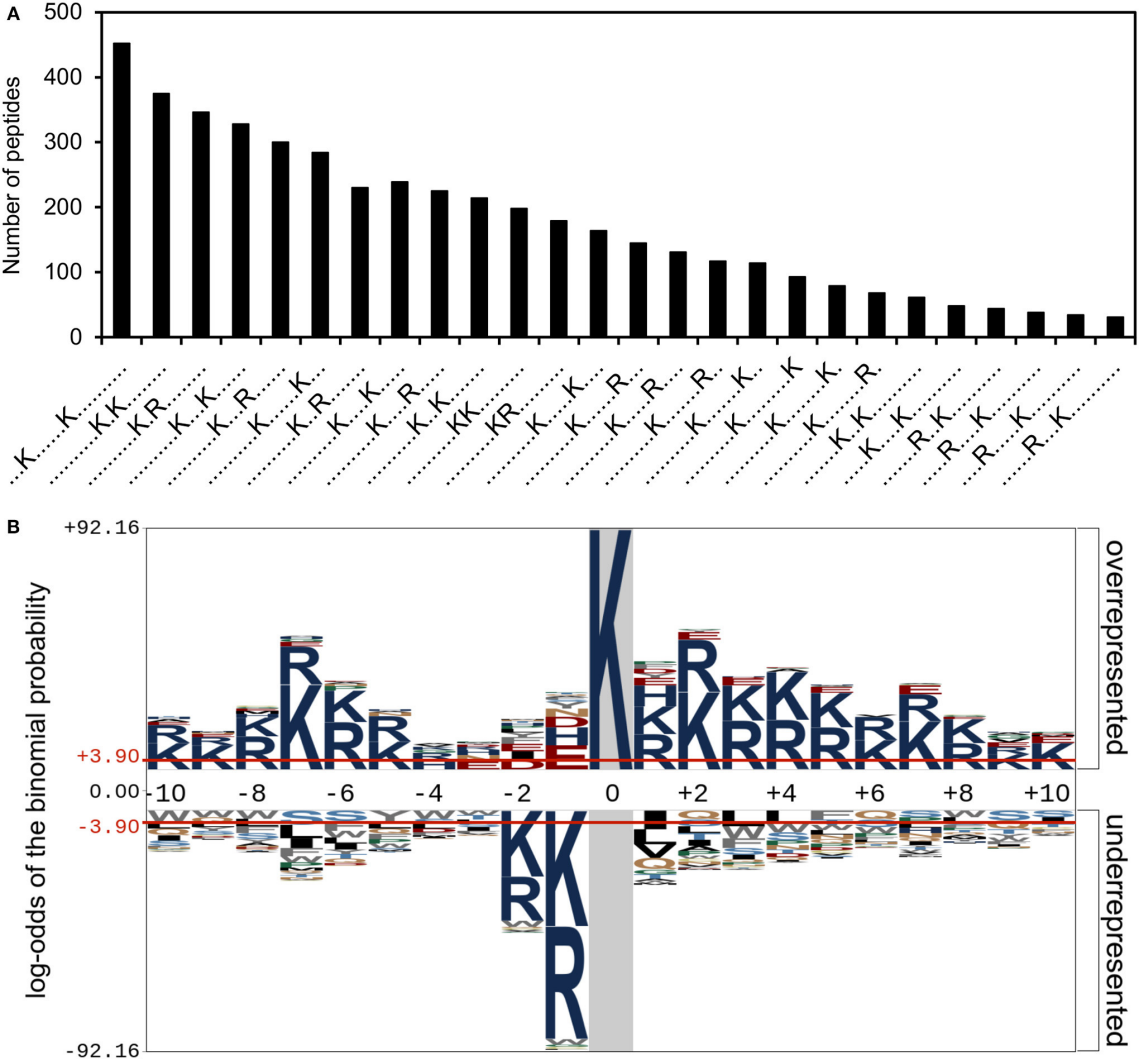
Sequence motifs surrounding acetylation sites. **(A)** Number of peptides containing significantly enriched motifs as identified using MotifX. **(B)** The significantly over- and under-represented amino acid residues surrounding the acetylation sites. The acetylated lysine is in position 0 and is shown shaded in gray.

We have shown above that there was no correlation between the number of acetylation sites in a protein and the number of lysine residues or the secondary structure. The propensity to be acetylated might also reside in a beneficial local environment such as a recognition site for a protein acetyltransferase and/or a chemical environment facilitating the transfer of the acetyl group from acetyl phosphate. We therefore analyzed the local environment around the acetylation sites (21 aa window) using the software Motif-X and pLogo (Figure 3). Using Motif-X we identified 26 significantly enriched sequence motifs. These were all variations of the acetylated lysine with a lysine or arginine residue. When considering the analysis of over- and under-represented amino acids in the sequence window, in agreement with the motifs, we saw an overrepresentation of lysine and arginine in positions −10 to −6 and +1 to +10. However, an underrepresentation of lysine/arginine in positions −2 and −1 was observed.

### Functional annotation and over-representation analysis of acetylated proteins

**Figure 4:**
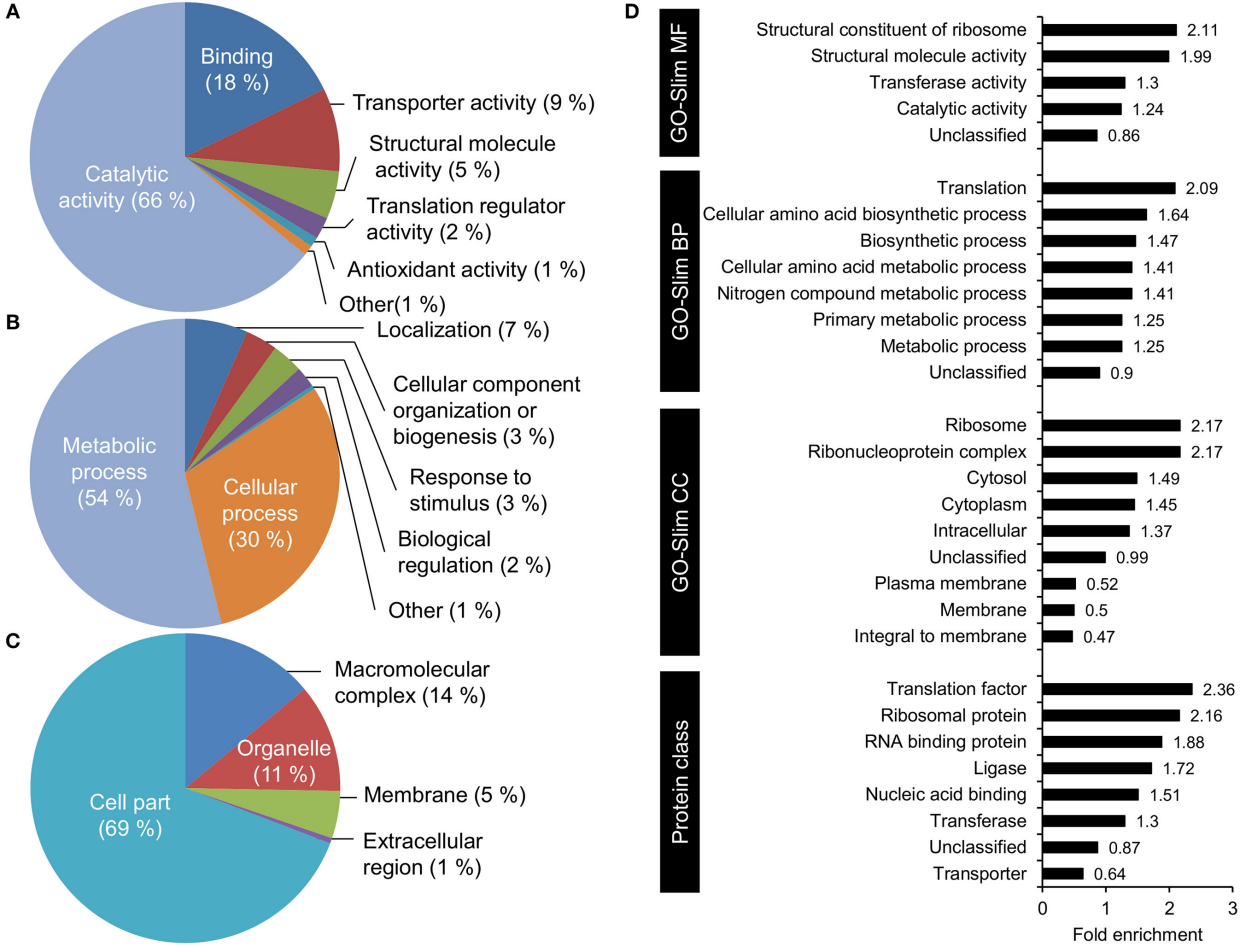
GO functional annotation and over-representation analysis of acetylated proteins. GO-Slim functional annotation with respect to **(A)** molecular function, **(B)** biological process, and **(C)** cellular component. **(D)** Functional over-representation based on the categories GO-Slim Molecular function, biological process, cellular component, and PANTHER protein class.

To understand what types of proteins were acetylated in *V. cholerae*, we used the PANTHER webpage tools (Mi et al., 2017) to perform a functional annotation of acetylated protein with GO-slim terms and an enrichment analysis of the associated GO terms (Figure 4). Functional annotation showed that within the GO term category molecular function, the majority of acetylated proteins were found to have catalytic activity (68%), while a substantial part had binding activity (18%). Small groups of proteins with transporter activity, structural roles, translational regulators, and other functions were observed. This is in line with the annotated biological processes terms that indicated roles in primarily metabolic processes.

The over-representation analysis demonstrated a significant (*P* < 0.05) over-representation of ribosomal structural proteins, and proteins involved in amino acid metabolism and translation (Figure 4D). An under-representation of unclassified proteins (proteins with no GO terms associated) was observed. With respect to cellular localization, there was an over-representation of cytosolic proteins and a subsequent under-representation of membrane proteins. When considering protein classes, proteins involved in protein synthesis, and nucleotide-binding proteins were enriched.

**Figure 5:**
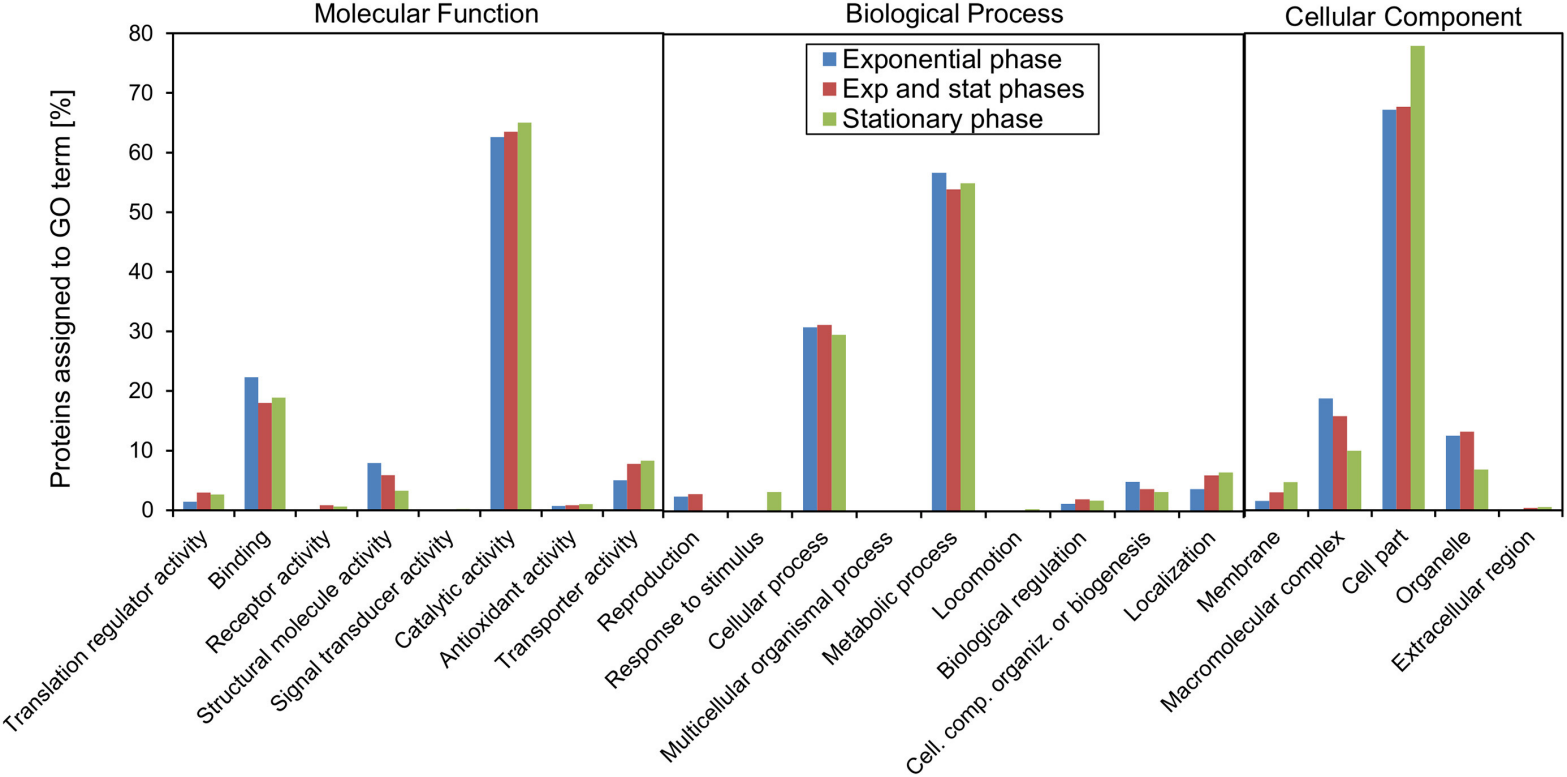
GO functional annotation in which the protein groups depend on the experimental condition in which the acetylation sites were detected. The three groups reflect whether a site was observed in only exponential growth phase, only stationary phase, or in both phases.

We also made the analysis taking into the account the growth stage where the acetylation event was detected in order to assess if there would be any global changes in the pattern (Figure 5). For the major terms, there was surprisingly little difference associated with growth phase. For some of the more specific molecular function terms related to regulatory processes, “translation regulatory activity,” “receptor activity,” “signal transducer activity,” and “transporter activity,” there was a higher fraction of proteins acetylated in stationary, or both phases. In contrast, the fraction of acetylated proteins assigned to the term “structural molecule activity” was highest in exponential phase and decreased toward stationary phase. When considering the biological function terms, the fraction of proteins associated with the terms “reproduction,” “cellular component organization or biogenesis” was highest in exponential phase and decreased toward stationary phase. The terms “response to stimulus,” biological regulation,” and “localization” on the hand increased in stationary phase.

**Figure 6:**
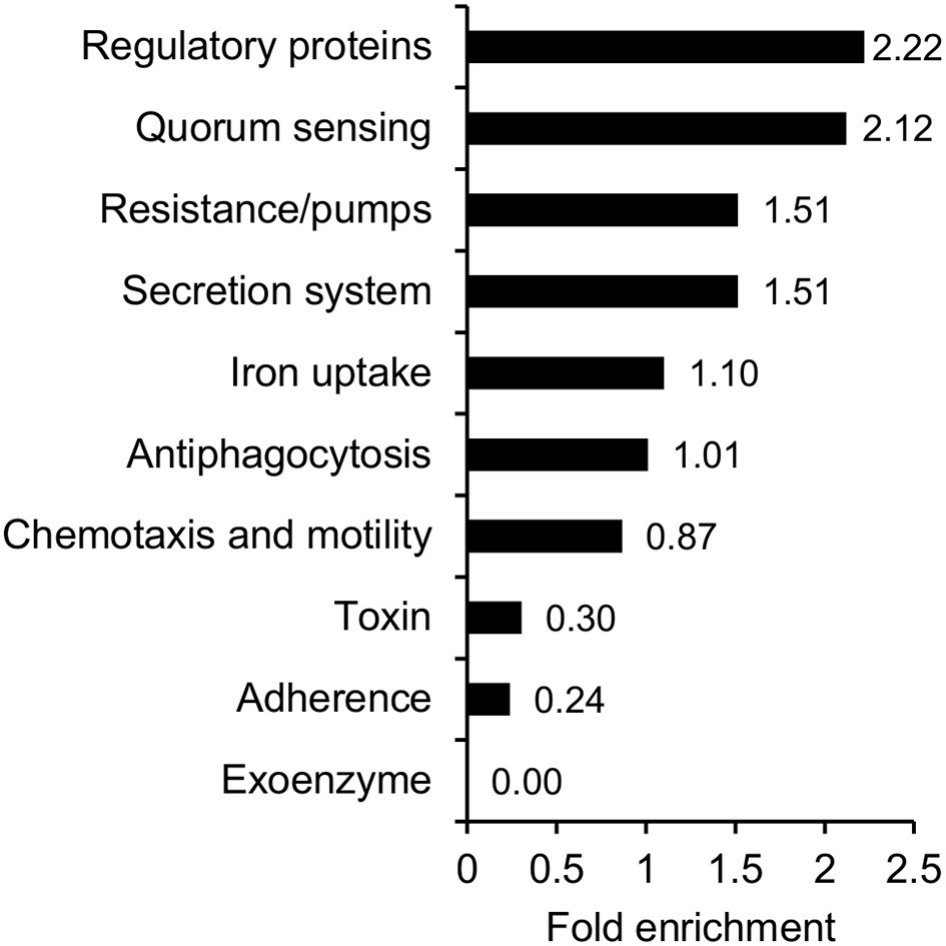
Acetylation of virulence factors. Functional over-representation based on the categories GO-Slim Molecular function, biological process, cellular component, and PANTHER protein class for the subset of proteins involved in virulence.

Considering the pathogenic nature of *V. cholerae*, it is of relevance to address the question, whether protein acetylation could play a role in pathogenicity. We therefore compiled a list of known virulence factors and performed an over-representation analysis on this subgroup (Figure 6, Supplementary Table 4). Our list of virulence factors comprised 203 proteins, and for these, we found 189 acetylation sites on 68 proteins. This corresponds to 33% of the virulence factors which is similar to the overall percentage of acetylated proteins. Of the 189 sites, 19 were acetylated only in exponential phase, 48 sites only in stationary phase, and the remaining 122 sites were acetylated in both conditions. The over-representation analysis indicated that preferentially regulatory proteins (mainly transcription factors and transcriptional regulators) and proteins involved in quorum sensing were acetylated.

### Structure examination of acetylation sites in virulence factors

**Figure 7:**
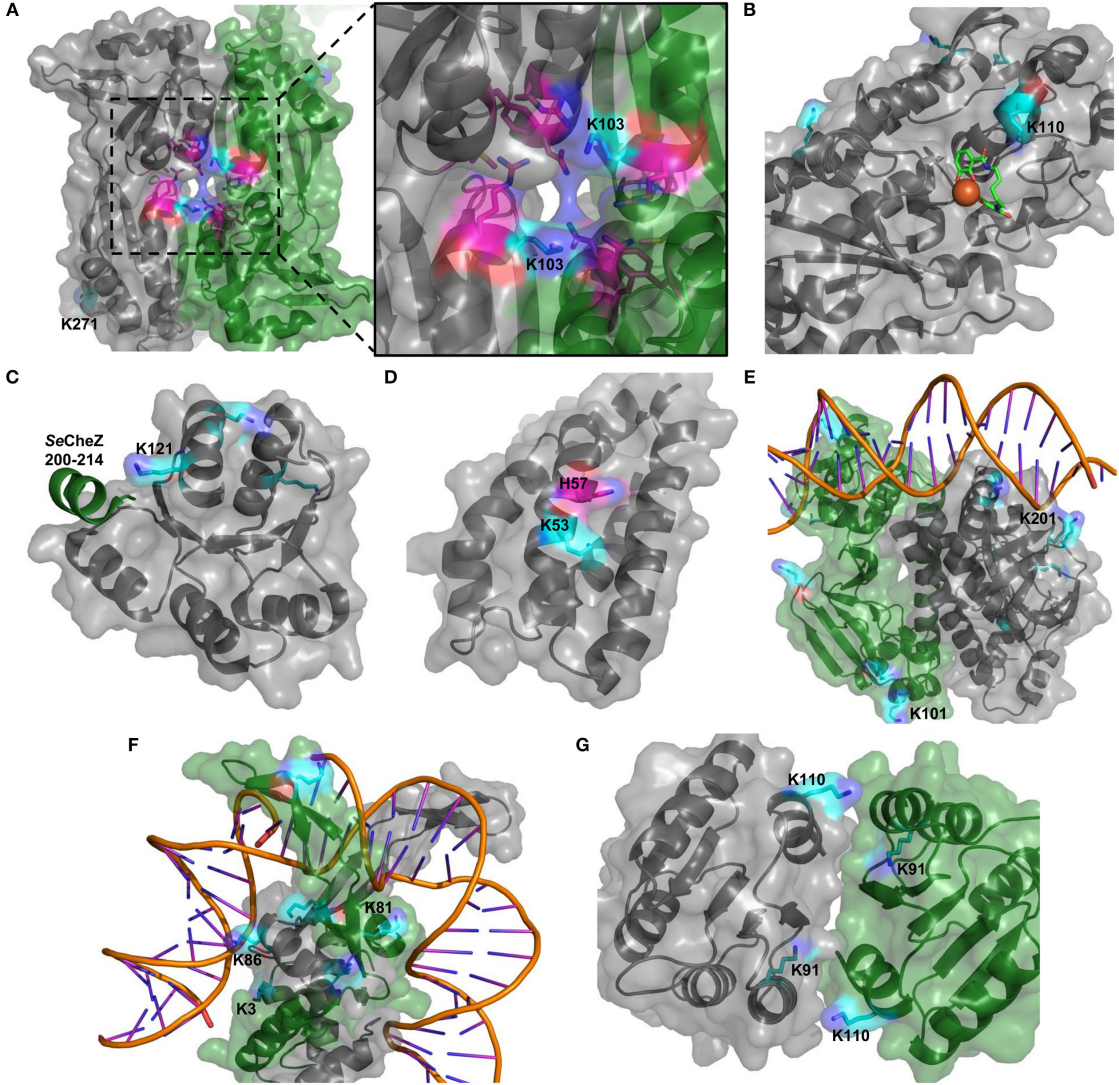
Structural analysis of acetylation sites in virulence factors. Structures of AphP **(A)**, VctP **(B)**, and CheY **(C)**, and homology models of LuxU **(D)**, CRP **(E)**, IhfA/B **(F)**, and PhoB **(G)** were evaluated. The proteins are depicted in gray or if two proteins in gray and green. Acetylated sites are shown in cyan and other relevant residues in purple (in **A** residues forming a binding site and in **D** the phosphorylation site).

For several of the virulence factors, the structure has been solved and based on these structures, we analyzed the positions of acetylated residues (Figure 7). The important transcriptional regulator AphB (KNH50088) was acetylated on K103 (stationary phase) and K271 (both exp. and stat. phases). Both residues are found far from the DNA binding site, but while K271 is located on the back relative to the dimerization interface, the K103 was in a position to potentially affect dimerization (Figure 7A). In the *Vibrio vulnificus* AphB homolog, K103 was shown to interact with an as yet unknown ligand (Park et al., 2017). Acetylation of vibriobactin transporter VctP (KNH52012) K110 was identified in both growth conditions. This residue is located in the vibriobactin binding pocket and in a position to possibly interact directly with vibriobactin (Figure 7B). The residue was previously suggested to form hydrogen bonds to the siderophore (Liu et al., 2012) and acetylation of this residue thus would be likely to affect the siderophore-binding of VctP. The *V. cholerae* chemotaxis response regulator CheY (KNH50695) was acetylated in both growth phases on K121 that is located in a position to affect interaction with CheZ (Figure 7C). CheY is likely to interact with FliM in the same interface (Biswas et al., 2013) and hence protein acetylation could affect motility. Substantiating this, another acetylated residue, K116, is known to affect switching of the flagellar motor (Hyakutake et al., 2005). To strengthen these observations that could indicate a regulatory potential of acetylation in virulence, we constructed homology models of additional proteins for which appropriate templates were available. The phosphorelay protein LuxU (KNH49903) that is phosphorylated on His-57 was found to be acetylated on K53 in stationary phase. This residue is in close vicinity to the phosphorylation site and acetylation could thus be expected to affect the phosphorylation process and/or the transfer of the phosphoryl moiety to LuxO thus affecting the virulence process (Figure 7D). The cAMP receptor protein (CRP)(KNH49720) is well-known to be an important transcriptional regulator of virulence gene regulation and biofilm formation of *V. cholerae* (Li et al., 2002; Liang et al., 2007; Fong and Yildiz, 2008). In this study, we observed that CRP was acetylated on six residues. Several acetylated residues were found close to the DNA binding site where especially the position of K202 that is acetylated in stationary phase could indicate a direct contact with the DNA molecule (Figure 7E). K101 is located immediately adjacent to activating region 2 through which it interacts with RNA polymerase (Won et al., 2009). Moreover, both subunits of integration host factor, IhfA (KNH49144) and IhfB (KNH50487) were acetylated on two and four residues, respectively. Ihf is a global transcriptional regulator that positively affects virulence gene expression in *V. cholerae* (Stonehouse et al., 2008). It was also reported that IHF is required for efficient transfer of integrative conjugative elements SXT and RP4 to and from *V. cholerae* (McLeod et al., 2006). Especially IhfA K86 (stationary phase) and IhfB K3 (both phases), and K81 (stationary phase) were found to be in relatively close vicinity to bound DNA (~5 Å) (Figure 7F). Finally, the two-component system response regulator PhoB (KNH49471) was acetylated on K91 (both phases) and K110 (exponential phase). Both residues, but in particular K91, are located in the dimerization interface and hence could affect dimerization and in turn activity of the response regulator (Figure 7G). In general, the Pho regulon in bacteria is not only involved in phosphate homeostasis but also plays an important role in stress response and virulence gene regulation (Chekabab et al., 2014).

In conclusion, the analysis demonstrated that several of the residues acetylated are in a position to potentially affect protein function of virulence factors in *V. cholerae*. Of note, many of the described acetylation events appear to be restricted to stationary phase.

### Known regulatory acetylation events are present in *V. cholerae*

It has been shown that acetylated lysine residues exhibit a substantial phylogenetic conservation (van Noort et al., 2012). We therefore wondered whether any of the characterized, regulatory acetylation events described in other bacteria would be conserved in *V. cholerae* proteins. We found several examples (Supplementary Figure 4) which indirectly suggests that the acetylation events also are regulatory in *V. cholerae*.

One of the best characterized acetylated proteins is acetyl coenzyme A synthetase. Its activity is regulated by inhibitory acetylation, and this process appears to be conserved from bacteria to humans (Starai et al., 2002; Schwer et al., 2006). The *V. cholerae* acetyl coenzyme A synthetase (KNH49371) was found acetylated, but not on the conserved regulatory site. Instead, we found that the structurally similar acetoacetyl coenzyme A synthetase (KNH51540) was acetylated on this site, indicating enzyme-dependent regulation of its activity. The *E. coli* tricarboxylic acid cycle enzyme malate dehydrogenase is acetylated on K99 and K140 which is known to increase its enzyme activity (Venkat et al., 2017). In our study, we have identified *V. cholerae* malate dehydrogenase (KNH48740) as acetylated on K140. S-adenosylmethionine synthetase catalyzes the formation of S-adenosyl methione that is an essential metabolite in all kingdoms of life (Fontecave et al., 2004). The *V. cholerae* S-adenosylmethionine synthetase (KNH48827) was shown to be acetylated on five residues. Three of these acetylation events (K4, K267, and K285) regulate the activity of the enzyme in *E. coli* (Sun et al., 2016). A homolog of the *Mycobacterium smegmatis* universal stress protein was identified in *V. cholerae* (KNH48736) that shared 23% identity with that of the *Mycobacterium smegmatis* enzyme. The *V. cholerae* universal stress protein was found to be acetylated on four lysines including K120 shown to be acetylated *in vivo* by protein acetyltransferase MSMEG_5458 in *M. smegmatis* (Nambi et al., 2010). The leucine-responsive regulatory protein Lrp (KNH50497), a global transcriptional regulator known to regulate up to 10% of the genome in *E. coli* (Cho et al., 2008) and virulence in *V. cholerae* (Lin et al., 2007), was found to be acetylated on four sites including the K36 that in *Salmonella typhimurium* was shown to regulate DNA binding *in vivo* (Qin et al., 2016).

In line with the reported high degree of phylogenetic conservation of acetylated lysine residues, we were able to identify several acetylation sites previously characterized in other bacteria. This suggests that at least a subset of regulatory acetylation events are evolutionary conserved, and points toward putative roles of protein acetylation in diverse cellular processes including metabolism, stress response, and transcriptional regulation in *V. cholerae*.

## Discussion

In this study we wanted to address the hypothesis that protein acetylation is an important regulatory modification in the pathogenic bacterium *V. cholerae*. Protein acetylation is considered a regulatory post translational modification resembling protein phosphorylation. A key feature of protein phosphorylation is that it is reversible via the action of kinases and phosphatases and hence can enable a fast response to a relevant stimulus. For acetylation, the modification of protein substrates can take place both enzymatically and non-enzymatically, but a deacetylase activity is necessary in order to assure reversibility of the post translational modification. In *V. cholerae* we identified 38 GNAT-domain containing proteins but it is well-known that many of these enzymes are likely to target alternate substrates (e.g., small molecules). Importantly, we identified both a putative NAD^+^-dependent sirtuin-like protein deacetylase and a putative NAD^+^-independent protein deacetylase. At present, there are no reports on regulation mediated by protein acetylation in *Vibrio* sp. but the presence of protein deacetylases clearly points to a regulatory function of protein acetylation in *V. cholerae*.

Having shown that protein acetylation is likely to be a regulatory post translational modification in *V. cholerae* we then wanted to address the global level of protein acetylation. We therefore performed a global protein acetylome study and detected a total of 3,402 acetylation sites on 1,240 acetylated proteins. Of the 3,402 sites, 259 sites were detected exclusively in exponential phase and 1137 site exclusively in stationary phase. This might reflect protein acetylation being dynamic, i.e., that the level of acetylation changes upon variation in growth stage. The overall number of acetylated proteins and sites detected here were significantly higher than that recorded for the related organism, *V. parahemolyticus*, where only 1,413 sites on 656 proteins were identified (Pan et al., 2014). This however might simply reflect a deeper coverage of the acetylome due to e.g., improved sensitivity of mass spectrometers.

We analyzed the sequence motifs surrounding the acetylation sites and observed an over-representation of different combinations of lysine and arginine and these residues were over-represented in the vicinity of the acetylation sites. In some studies, similar patterns were suggested to be acetyltransferase recognition sites, but this explanation is not easily reconciled with the fact that majority of acetylation events are a consequence of chemical acetylation by acetyl phosphate (Weinert et al., 2013). Consequently, it should appear more likely that these positively charged residues could favor the transfer of acetyl from acetyl phosphate. An alternative explanation resides in the fact that acetylated lysine residues are resistant to the proteases trypsin and LysC used in the proteomic workflow. The absence of an arginine or an unmodified lysine in the vicinity of the acetylated lysine would thus lead to a long peptide that would be more difficult to detect by MS (Baldwin, 2004).

When considering the types of enzymes that are acetylated, it is clear from the data that essentially all parts of the cell could be potentially regulated by protein acetylation. Our analysis indicated an over-representation of acetylation of proteins involved in metabolic and cellular processes. This is quite similar to what has been shown in the related organism *V. parahemolyticus* (Pan et al., 2014). In addition to performing over-expression analyses based on GO terms as is the standard approach, we also compiled a list of known and putative virulence factors in *V. cholerae*. This list comprised 203 proteins, and of these, 33% were found to be acetylated. For this important group of proteins, we saw that several global transcriptional regulators as well as transcription factors more specifically regulating virulence, Type VI secretion factors and proteins involved in quorum sensing were acetylated.

Expression of virulence genes encoding the major virulence determinants cholera toxin, toxin coregulated pilus, and biofilm formation is under control of a complex regulatory cascade involving AphAB (Kovacikova et al., 2010; Yang et al., 2010; Rutherford et al., 2011), TcpP/H, and ToxT (Kovacikova and Skorupski, 2000; Yang et al., 2013), and this cascade is modulated by additional transcriptional regulators H-NS (Nye et al., 2000), HapR (Liu et al., 2008; Rutherford et al., 2011), CRP (Skorupski and Taylor, 1997), Lrp (Lin et al., 2007), and PhoB (Pratt et al., 2010) that aids to coordinate virulence gene transcription. Interestingly, a majority of these transcriptional regulators (AphB, TcpP, H-NS, Lrp, PhoB, and CRP) were found to be acetylated in this study providing the possibility that a thus far uncharacterized, novel layer of regulation of this important phenomenon could exist. In addition, we performed a structural analysis for three of these enzymes, AphB, CRP, and PhoB, and found acetylation sites that were in location to regulate protein function. Dimerization of AphB is required for the expression of the key virulence regulator TcpP, which leads to the activation of virulence factor production (Liu et al., 2011). In *E. coli*, phosphorylation-mediated dimerization of PhoB is required for binding of PhoB to tandem DNA-binding sites and thus regulation of transcription (Creager-Allen et al., 2013). In this study, the acetylation sites of AphB and PhoB were observed to be located in protein-protein interaction interfaces and thus could influence AphB dimerization and interaction of PhoB with its multiple interaction partners. For CRP, acetylation could affect interaction with its target DNA as known for histone acetylation that leads to loss of affinity for DNA (Chen et al., 2015). By analyzing other virulence factors, we found more examples where lysine acetylation could be expected to affect protein-protein interaction and DNA binding. It is noteworthy that most of the acetylation sites for which a potential regulatory role could be found, were identified only in stationary phase. It has previously been shown that virulence gene expression in general is very low in LB medium in the early stages of growth (Kanjilal et al., 2010), which might point to an explanation as to why potential virulence regulation was mainly identified in stationary phase. It will require an in-depth study in a more appropriate pathogenesis model system to prove a role for acetylation in regulating the infection process, but certainly the data presented here could indicate such a role.

In conclusion, we have demonstrated that protein acetylation is abundant in *V. cholerae* and our analyses indicate that it could be an important means of regulation in several cellular processes, including virulence. This first report of the global protein acetylome in *V. cholerae* provides a foundation for deciphering the functional roles of protein acetylation-based regulation in this organism.

## Author contributions

CJ, VR, and IM designed experiments; CJ, VR, and AS performed the experiments; CJ, VR, ML, AS, ÅS, SW, and IM performed the data analysis; CJ drafted the manuscript; All authors revised the manuscript and approved the final version.

### Conflict of interest statement

The authors declare that the research was conducted in the absence of any commercial or financial relationships that could be construed as a potential conflict of interest.
