# Supplementary material for: The Global Acetylome of the Human Pathogen Vibrio cholerae V52 Reveals Lysine Acetylation of Major Transcriptional Regulators
Source: Front Cell Infect Microbiol. 2018 Jan 11;7:537. doi: 10.3389/fcimb.2017.00537 (PMC5768985; doi:10.3389/fcimb.2017.00537)
Supplement: Supplementary file 1 [file Table1.PDF]

Supplementary Table 1. Identification of potential protein deacetylases in *Vibrio cholerae*. The presence of protein deacetylase-related domains in the *V. cholerae* genome was evaluated using the NCBI Conserved Domains search tool. The output for the 2 identified putative protein deacetylases is shown.

Accession	Annotation	Hit type	PSSM-ID	From	To	E-Value	Bitscore	Accession	Short name	Incomplete	Superfamily
KNH50718	histone deacetylase/ AcuC/AphA family protein	**specific**	**181564**	**422**	**803**	**0**	**737.736**	**PRK08849**	**PRK08849**	**-**	**cl21454**
		**superfamily**	**328723**	**422**	**803**	**0**	**737.736**	**cl21454**	**NADB_Rossmann superfamily**	**-**	**-**
		**specific**	**273913**	**440**	**802**	**1.86E-134**	**404.278**	**TIGR01988**	**Ubi-OHases**	**-**	**cl27552**
		**superfamily**	**332373**	**440**	**802**	**1.86E-134**	**404.278**	**cl27552**	**FAD_binding_3 superfamily**	**-**	**-**
		**specific**	**212519**	**19**	**298**	**6.18E-133**	**396.098**	**cd09993**	**HDAC_classIV**	**-**	**cl17011**
		**superfamily**	**327367**	**19**	**298**	**6.18E-133**	**396.098**	**cl17011**	**Arginase_HDAC superfamily**	**-**	**-**
		**specific**	**223727**	**440**	**803**	**7.56E-106**	**330.129**	**COG0654**	**UbiH**	**-**	**cl27552**
		**specific**	**223201**	**1**	**286**	**3.03E-86**	**276.948**	**COG0123**	**AcuC**	**-**	**cl17011**
		**specific**	**307135**	**19**	**298**	**1.14E-60**	**207.072**	**pfam00850**	**Hist_deacetyl**	**-**	**cl17011**
		**non-specific**	**240251**	**113**	**283**	**1.54E-15**	**79.4695**	**PTZ00063**	**PTZ00063**	**NC**	**cl26608**
		**superfamily**	**240251**	**113**	**283**	**1.54E-15**	**79.4695**	**cl26608**	**PTZ00063 superfamily**	**NC**	**-**
		**non-specific**	**307580**	**573**	**752**	**1.17E-10**	**63.8739**	**pfam01494**	**FAD_binding_3**	**N**	**cl27552**
KNH50969	NAD-dependent deacetylase	**specific**	**234777**	**5**	**236**	**2.85E-131**	**369.893**	**PRK00481**	**PRK00481**	**-**	**cl00195**
		**superfamily**	**320810**	**5**	**236**	**2.85E-131**	**369.893**	**cl00195**	**SIR2 superfamily**	**-**	**-**
		**specific**	**238703**	**6**	**231**	**1.11E-117**	**334.944**	**cd01412**	**SIRT5_Af1_CobB**	**-**	**cl00195**
		**specific**	**223915**	**6**	**238**	**1.70E-104**	**302.654**	**COG0846**	**SIR2**	**-**	**cl00195**
		**non-specific**	**280333**	**13**	**186**	**5.34E-70**	**212.493**	**pfam02146**	**SIR2**	**-**	**cl00195**
