# Supplementary material for: The Global Acetylome of the Human Pathogen Vibrio cholerae V52 Reveals Lysine Acetylation of Major Transcriptional Regulators
Source: Front Cell Infect Microbiol. 2018 Jan 11;7:537. doi: 10.3389/fcimb.2017.00537 (PMC5768985; doi:10.3389/fcimb.2017.00537)
Supplement: Supplementary file 2 [file Table2.PDF]

Supplementary Table 2. Identificiation of potential acetyltransferases in *Vibrio cholerae*. The presence of GNAT domains in the *V. cholerae* genome was evaluated using the NCBI Conserved Domains search tool. The output for the 38 identified GNAT-domain containing proteins is shown.

Accession	Annotation	Hit type	PSSM-ID	From	To	E-Value	Bitscore	Accession	Short name	Incomplete	Superfamily
KNH51167	GNAT family Acetyltransferase	non-specific	236715	3	122	3.20E-19	77.8003	PRK10562	PRK10562	-	cl17182
		**superfamily**	**327402**	**3**	**122**	**3.20E-19**	**77.8003**	**cl17182**	**NAT_SF superfamily**	**-**	**-**
		**specific**	**306954**	**11**	**119**	**4.97E-15**	**66.3794**	**pfam00583**	**Acetyltransf_1**	**-**	**cl17182**
		**non-specific**	**273701**	**55**	**133**	**2.45E-09**	**51.5607**	**TIGR01575**	**rimI**	**N**	**cl26092**
		**superfamily**	**330913**	**55**	**133**	**2.45E-09**	**51.5607**	**cl26092**	**Acetyltransf_10 superfamily**	**N**	**-**
		**specific**	**223532**	**1**	**133**	**6.43E-09**	**51.5358**	**COG0456**	**RimI**	**-**	**cl26092**
		**specific**	**173926**	**48**	**90**	**1.55E-07**	**45.3445**	**cd04301**	**NAT_SF**	**C**	**cl17182**
KNH48625	hypothetical protein	**specific**	**316066**	**38**	**111**	**8.61E-09**	**49.3653**	**pfam13508**	**Acetyltransf_7**	**-**	**cl17182**
		**superfamily**	**327402**	**38**	**111**	**8.61E-09**	**49.3653**	**cl17182**	**NAT_SF superfamily**	**-**	**-**
		**specific**	**173926**	**42**	**91**	**3.19E-05**	**39.5665**	**cd04301**	**NAT_SF**	**-**	**cl17182**
		non-specific	182373	71	119	0.005929	34.8192	PRK10314	PRK10314	N	cl17182
KNH48790	hemolysin	superfamily	327403	1	287	1.61E-100	306.632	cl17185	LPLAT superfamily	-	-
		specific	153248	52	261	6.71E-77	242.538	cd07986	LPLAT_ACT14924-like	-	cl17185
		**specific**	**316007**	**329**	**430**	**1.33E-31**	**117.72**	**pfam13444**	**Acetyltransf_5**	**-**	**cl17185**
		superfamily	327403	329	430	1.33E-31	117.72	cl17185	LPLAT superfamily	-	-
		specific	214724	76	199	6.34E-16	73.9286	smart00563	PlsC	-	cl17185
		non-specific	279841	62	196	6.84E-06	45.7323	pfam01553	Acyltransferase	-	cl17185
		non-specific	225717	330	567	1.37E-05	47.0075	COG3176	COG3176	N	cl17185
KNH49141	histone acetyltransferase	**specific**	**306954**	**48**	**137**	**7.90E-19**	**76.7798**	**pfam00583**	**Acetyltransf_1**	**N**	**cl17182**
		**superfamily**	**327402**	**48**	**137**	**7.90E-19**	**76.7798**	**cl17182**	**NAT_SF superfamily**	**N**	**-**
		**specific**	**173926**	**56**	**120**	**2.95E-12**	**58.056**	**cd04301**	**NAT_SF**	**-**	**cl17182**
		**non-specific**	**273701**	**51**	**138**	**5.57E-11**	**56.5683**	**TIGR01575**	**rimI**	**N**	**cl26092**
		**superfamily**	**330913**	**51**	**138**	**5.57E-11**	**56.5683**	**cl26092**	**Acetyltransf_10 superfamily**	**N**	**-**
		**non-specific**	**224167**	**50**	**139**	**4.80E-10**	**54.6312**	**COG1246**	**ArgA**	**NC**	**cl17182**
		non-specific	236088	56	113	3.89E-06	44.0297	PRK07757	PRK07757	NC	cl17182

KNH49221	ribosomal-protein-S5p-alanine acetyltransferase	non-specific	182749	17	189	3.76E-67	203.432	PRK10809	PRK10809	-	cl17182
		**superfamily**	**327402**	**17**	**189**	**3.76E-67**	**203.432**	**cl17182**	**NAT_SF superfamily**	**-**	**-**
		specific	224584	17	191	2.17E-36	125.061	COG1670	RimL	-	cl26094
		**superfamily**	**330915**	**17**	**191**	**2.17E-36**	**125.061**	**cl26094**	**Acetyltransf_3 superfamily**	**-**	**-**
		**specific**	**315878**	**17**	**161**	**4.93E-21**	**83.9334**	**pfam13302**	**Acetyltransf_3**	**-**	**cl26094**
KNH49250	histone acetyltransferase	**specific**	**306954**	**23**	**146**	**7.03E-17**	**71.387**	**pfam00583**	**Acetyltransf_1**	**-**	**cl17182**
		**superfamily**	**327402**	**23**	**146**	**7.03E-17**	**71.387**	**cl17182**	**NAT_SF superfamily**	**-**	**-**
		**non-specific**	**273701**	**35**	**146**	**1.62E-14**	**65.8131**	**TIGR01575**	**rimI**	**-**	**cl26092**
		**superfamily**	**330913**	**35**	**146**	**1.62E-14**	**65.8131**	**cl26092**	**Acetyltransf_10 superfamily**	**-**	**-**
		specific	223532	46	157	3.58E-13	63.477	COG0456	RimI	N	cl26092
		**specific**	**173926**	**59**	**129**	**4.40E-11**	**54.9744**	**cd04301**	**NAT_SF**	**-**	**cl17182**
		non-specific	236088	60	128	2.66E-07	47.1113	PRK07757	PRK07757	NC	cl17182
KNH50511	ribosomal-protein-serine acetyltransferase	**specific**	**315878**	**11**	**152**	**1.42E-32**	**113.209**	**pfam13302**	**Acetyltransf_3**	**-**	**cl26094**
		**superfamily**	**330915**	**11**	**152**	**1.42E-32**	**113.209**	**cl26094**	**Acetyltransf_3 superfamily**	**-**	**-**
		specific	224584	6	177	1.73E-29	106.956	COG1670	RimL	-	cl26094
		non-specific	182270	7	151	4.10E-11	58.6179	PRK10151	PRK10151	-	cl17182
		**superfamily**	**327402**	**7**	**151**	**4.10E-11**	**58.6179**	**cl17182**	**NAT_SF superfamily**	**-**	**-**
KNH51829	acetyltransferase	**specific**	**225261**	**1**	**93**	**7.84E-32**	**106.658**	**COG2388**	**YidJ**	**-**	**cl17182**
		**superfamily**	**327402**	**1**	**93**	**7.84E-32**	**106.658**	**cl17182**	**NAT_SF superfamily**	**-**	**-**
		specific	317008	14	90	1.46E-27	95.2764	pfam14542	Acetyltransf_CG	-	cl17182
KNH52046	acetyltransferase	specific	225695	1	177	3.10E-53	167.077	COG3153	yhbS	-	cl17182
		**superfamily**	**327402**	**1**	**177**	**3.10E-53**	**167.077**	**cl17182**	**NAT_SF superfamily**	**-**	**-**
		**specific**	**316066**	**49**	**132**	**7.22E-11**	**55.5285**	**pfam13508**	**Acetyltransf_7**	**-**	**cl17182**
		**specific**	**173926**	**53**	**120**	**3.57E-08**	**48.0409**	**cd04301**	**NAT_SF**	**-**	**cl17182**
		**superfamily**	**330913**	**53**	**138**	**1.90E-07**	**47.6462**	**cl26092**	**Acetyltransf_10 superfamily**	**N**	**-**
		non-specific	236088	54	137	9.51E-05	40.5629	PRK07757	PRK07757	N	cl17182
		non-specific	132489	86	132	0.000827	38.5364	TIGR03448	mycothiol_MshD	N	cl26092
KNH51231	hypothetical protein	**specific**	**316217**	**36**	**158**	**7.23E-20**	**79.6178**	**pfam13673**	**Acetyltransf_10**	**-**	**cl26092**
		**superfamily**	**330913**	**36**	**158**	**7.23E-20**	**79.6178**	**cl26092**	**Acetyltransf_10 superfamily**	**-**	**-**
		specific	224584	3	157	2.87E-18	76.9106	COG1670	RimL	-	cl26094
		**superfamily**	**330915**	**3**	**157**	**2.87E-18**	**76.9106**	**cl26094**	**Acetyltransf_3 superfamily**	**-**	**-**

		**superfamily**	**327402**	**14**	**132**	**1.07E-12**	**60.6014**	**cl17182**	**NAT_SF superfamily**	-	-
		**non-specific**	**273701**	**31**	**135**	**7.06E-08**	**48.0939**	**TIGR01575**	**rimI**	-	**cl26092**
		**specific**	**173926**	**53**	**104**	**0.000223**	**37.2553**	**cd04301**	**NAT_SF**	-	**cl17182**
		non-specific	181904	85	136	0.000666	37.6014	PRK09491	rimI	N	cl17182
KNH51251	acetyltransferase	**specific**	**316066**	**88**	**154**	**5.24E-08**	**47.8245**	**pfam13508**	**Acetyltransf_7**	**N**	**cl17182**
		**superfamily**	**327402**	**88**	**154**	**5.24E-08**	**47.8245**	**cl17182**	**NAT_SF superfamily**	**N**	-
		**superfamily**	**330913**	**96**	**169**	**3.26E-07**	**46.8758**	**cl26092**	**Acetyltransf_10 superfamily**	**N**	-
		non-specific	225695	92	158	2.25E-05	42.272	COG3153	yhbS	NC	cl17182
		**non-specific**	**273701**	**96**	**154**	**0.001209**	**36.9231**	**TIGR01575**	**rimI**	**N**	**cl26092**
		non-specific	140351	101	162	0.004836	35.5883	PTZ00330	PTZ00330	N	cl17182
KNH51324	acetyltransferase	specific	223972	1	637	0	671.76	COG1042	ACCS	-	cl26126
		superfamily	330947	1	637	0	671.76	cl26126	Ligase_CoA superfamily	-	-
		non-specific	131764	4	453	8.19E-145	435.971	TIGR02717	AcCoA-syn-alpha	-	cl26126
		specific	316106	493	706	3.09E-87	277.033	pfam13549	ATP-grasp_5	-	cl17255
		superfamily	327410	493	706	3.09E-87	277.033	cl17255	CPSase_L_D2 superfamily	-	-
		superfamily	328723	11	138	3.69E-27	106.452	cl21454	NADB_Rossmann superfamily	-	-
		specific	214881	7	100	1.28E-14	70.2314	smart00881	CoA_binding	-	cl21454
		**specific**	**306954**	**762**	**875**	**1.24E-09**	**56.3642**	**pfam00583**	**Acetyltransf_1**	-	**cl17182**
		**superfamily**	**327402**	**762**	**875**	**1.24E-09**	**56.3642**	**cl17182**	**NAT_SF superfamily**	-	-
		non-specific	224168	820	875	1.11E-05	46.5416	COG1247	YncA	N	cl26093
		**superfamily**	**330914**	**820**	**875**	**1.11E-05**	**46.5416**	**cl26093**	**Acetyltransf_4 superfamily**	**N**	-
		**superfamily**	**330913**	**736**	**875**	**9.04E-05**	**43.8319**	**cl26092**	**Acetyltransf_10 superfamily**	-	-
		**specific**	**173926**	**793**	**855**	**0.003802**	**36.4849**	**cd04301**	**NAT_SF**	-	**cl17182**
		**non-specific**	**273701**	**822**	**887**	**0.008508**	**37.3083**	**TIGR01575**	**rimI**	**N**	**cl26092**
KNH51486	acetyltransferase	specific	226489	1	171	1.42E-83	243.439	COG3981	COG3981	-	cl17182
		**superfamily**	**327402**	**1**	**171**	**1.42E-83**	**243.439**	**cl17182**	**NAT_SF superfamily**	-	-
		**specific**	**315878**	**3**	**148**	**4.45E-10**	**54.6582**	**pfam13302**	**Acetyltransf_3**	-	**cl26094**
		**superfamily**	**330915**	**3**	**148**	**4.45E-10**	**54.6582**	**cl26094**	**Acetyltransf_3 superfamily**	-	-
		**superfamily**	**330913**	**72**	**139**	**4.43E-05**	**41.5207**	**cl26092**	**Acetyltransf_10 superfamily**	**NC**	-
		**specific**	**173926**	**65**	**131**	**4.86E-05**	**39.1813**	**cd04301**	**NAT_SF**	-	**cl17182**

		non-specific	132489	64	145	0.000195	40.4624	TIGR03448	mycothiol_MshD	N	cl26092
		non-specific	236088	100	130	0.001116	37.4813	PRK07757	PRK07757	NC	cl17182
KNH51647	spermidine N1-acetyltransferase	non-specific	237916	3	171	6.63E-74	219.668	PRK15130	PRK15130	-	cl17182
		**superfamily**	**327402**	**3**	**171**	**6.63E-74**	**219.668**	**cl17182**	**NAT_SF superfamily**	**-**	**-**
		specific	224584	1	173	4.62E-33	115.816	COG1670	RimL	-	cl26094
		**superfamily**	**330915**	**1**	**173**	**4.62E-33**	**115.816**	**cl26094**	**Acetyltransf_3 superfamily**	**-**	**-**
		**specific**	**315878**	**5**	**140**	**1.92E-18**	**76.6146**	**pfam13302**	**Acetyltransf_3**	**-**	**cl26094**
		**non-specific**	**274661**	**48**	**162**	**5.46E-12**	**60.0651**	**TIGR03585**	**PseH**	**N**	**cl26094**
		**specific**	**173926**	**58**	**122**	**1.75E-05**	**40.7221**	**cd04301**	**NAT_SF**	**-**	**cl17182**
KNH51190	hypothetical protein	specific	224584	1	178	7.80E-36	123.135	COG1670	RimL	-	cl26094
		**superfamily**	**330915**	**1**	**178**	**7.80E-36**	**123.135**	**cl26094**	**Acetyltransf_3 superfamily**	**-**	**-**
		**specific**	**315878**	**8**	**147**	**1.12E-26**	**98.1858**	**pfam13302**	**Acetyltransf_3**	**-**	**cl26094**
		**superfamily**	**327402**	**67**	**146**	**2.68E-09**	**52.127**	**cl17182**	**NAT_SF superfamily**	**N**	**-**
		non-specific	237916	4	177	3.59E-08	50.5655	PRK15130	PRK15130	-	cl17182
		**non-specific**	**274661**	**8**	**156**	**0.000105**	**40.4199**	**TIGR03585**	**PseH**	**-**	**cl26094**
		**specific**	**173926**	**72**	**129**	**0.006892**	**33.4033**	**cd04301**	**NAT_SF**	**-**	**cl17182**
KNH51238	histone acetyltransferase	**specific**	**306954**	**32**	**133**	**4.84E-12**	**58.6754**	**pfam00583**	**Acetyltransf_1**	**-**	**cl17182**
		**superfamily**	**327402**	**32**	**133**	**4.84E-12**	**58.6754**	**cl17182**	**NAT_SF superfamily**	**-**	**-**
		specific	223532	1	144	7.75E-09	51.5358	COG0456	RimI	-	cl26092
		**superfamily**	**330913**	**1**	**144**	**7.75E-09**	**51.5358**	**cl26092**	**Acetyltransf_10 superfamily**	**-**	**-**
		**non-specific**	**273701**	**51**	**144**	**4.29E-08**	**48.8643**	**TIGR01575**	**rimI**	**N**	**cl26092**
		**specific**	**173926**	**55**	**116**	**9.12E-07**	**43.4185**	**cd04301**	**NAT_SF**	**-**	**cl17182**
		non-specific	182266	5	142	0.000155	39.1293	PRK10146	PRK10146	-	cl17182
KNH48577	hypothetical protein	**specific**	**316066**	**39**	**144**	**3.17E-08**	**48.2097**	**pfam13508**	**Acetyltransf_7**	**-**	**cl17182**
		**superfamily**	**327402**	**39**	**144**	**3.17E-08**	**48.2097**	**cl17182**	**NAT_SF superfamily**	**-**	**-**
		**superfamily**	**330913**	**22**	**145**	**4.15E-08**	**48.8018**	**cl26092**	**Acetyltransf_10 superfamily**	**-**	**-**
		non-specific	223532	88	144	4.23E-05	41.5207	COG0456	RimI	N	cl26092
KNH48605	acetyltransferase	non-specific	235142	1	135	2.88E-24	90.7582	PRK03624	PRK03624	-	cl26092
		**superfamily**	**330913**	**1**	**135**	**2.88E-24**	**90.7582**	**cl26092**	**Acetyltransf_10 superfamily**	**-**	**-**
		specific	223532	1	141	2.18E-20	81.9666	COG0456	RimI	-	cl26092
		**specific**	**306954**	**19**	**123**	**4.59E-16**	**69.0758**	**pfam00583**	**Acetyltransf_1**	**-**	**cl17182**

		**superfamily**	**327402**	**19**	**123**	**4.59E-16**	**69.0758**	**cl17182**	**NAT_SF superfamily**	**-**	**-**
		**specific**	**173926**	**47**	**107**	**5.97E-10**	**51.8928**	**cd04301**	**NAT_SF**	**-**	**cl17182**
		**non-specific**	**273701**	**49**	**132**	**2.21E-09**	**51.9459**	**TIGR01575**	**rimI**	**N**	**cl26092**
KNH48920	acetyltransferase	specific	225695	1	167	9.58E-69	205.597	COG3153	yhbS	-	cl17182
		**superfamily**	**327402**	**1**	**167**	**9.58E-69**	**205.597**	**cl17182**	**NAT_SF superfamily**	**-**	**-**
		**specific**	**316066**	**44**	**123**	**1.28E-10**	**54.7581**	**pfam13508**	**Acetyltransf_7**	**-**	**cl17182**
		non-specific	236088	1	130	9.80E-08	48.652	PRK07757	PRK07757	-	cl17182
		**specific**	**173926**	**44**	**110**	**1.86E-07**	**45.7297**	**cd04301**	**NAT_SF**	**-**	**cl17182**
		**superfamily**	**330913**	**1**	**127**	**2.48E-06**	**44.9875**	**cl26092**	**Acetyltransf_10 superfamily**	**-**	**-**
		**non-specific**	**273701**	**35**	**127**	**5.93E-05**	**40.3899**	**TIGR01575**	**rimI**	**-**	**cl26092**
KNH48918	ribosomal-protein-alanine acetyltransferase	non-specific	181904	1	144	2.39E-48	152.391	PRK09491	rimI	-	cl17182
		**superfamily**	**327402**	**1**	**144**	**2.39E-48**	**152.391**	**cl17182**	**NAT_SF superfamily**	**-**	**-**
		**non-specific**	**273701**	**12**	**144**	**5.70E-46**	**145.935**	**TIGR01575**	**rimI**	**-**	**cl26092**
		**superfamily**	**330913**	**12**	**144**	**5.70E-46**	**145.935**	**cl26092**	**Acetyltransf_10 superfamily**	**-**	**-**
		specific	223532	1	147	1.82E-24	92.367	COG0456	RimI	-	cl26092
		**specific**	**306954**	**13**	**121**	**7.72E-16**	**68.6906**	**pfam00583**	**Acetyltransf_1**	**-**	**cl17182**
		**specific**	**173926**	**45**	**104**	**7.00E-08**	**46.5001**	**cd04301**	**NAT_SF**	**-**	**cl17182**
KNH49776	galactoside O-acetyltransferase	specific	312863	157	297	5.55E-73	220.939	pfam09500	YiiD_C	-	cl00509
		superfamily	321012	157	297	5.55E-73	220.939	cl00509	hot_dog superfamily	-	-
		non-specific	131500	164	297	2.35E-51	165.604	TIGR02447	yiiD_Cterm	-	cl00509
		**specific**	**306954**	**13**	**122**	**1.37E-09**	**54.8234**	**pfam00583**	**Acetyltransf_1**	**-**	**cl17182**
		**superfamily**	**327402**	**13**	**122**	**1.37E-09**	**54.8234**	**cl17182**	**NAT_SF superfamily**	**-**	**-**
		**superfamily**	**330913**	**58**	**144**	**1.53E-09**	**54.965**	**cl26092**	**Acetyltransf_10 superfamily**	**N**	**-**
		non-specific	140351	67	125	9.75E-06	44.4479	PTZ00330	PTZ00330	N	cl17182
		**non-specific**	**225064**	**35**	**146**	**3.84E-05**	**43.0878**	**COG2153**	**ElaA**	**N**	**cl28579**
		**superfamily**	**225064**	**35**	**146**	**3.84E-05**	**43.0878**	**cl28579**	**ElaA superfamily**	**N**	**-**
		**non-specific**	**273701**	**63**	**128**	**0.000338**	**39.6195**	**TIGR01575**	**rimI**	**N**	**cl26092**
		non-specific	224961	158	297	0.001007	38.5173	COG2050	PaaI	-	cl00509
		**specific**	**173926**	**56**	**108**	**0.003425**	**35.3293**	**cd04301**	**NAT_SF**	**-**	**cl17182**
KNH48589	acetyltransferase	specific	224584	1	168	9.99E-16	70.7474	COG1670	RimL	-	cl26094
		**superfamily**	**330915**	**1**	**168**	**9.99E-16**	**70.7474**	**cl26094**	**Acetyltransf_3 superfamily**	**-**	**-**
		**specific**	**306954**	**33**	**143**	**9.95E-13**	**60.9866**	**pfam00583**	**Acetyltransf_1**	**-**	**cl17182**

		**superfamily**	**327402**	**33**	**143**	**9.95E-13**	**60.9866**	**cl17182**	**NAT_SF superfamily**	**-**	**-**
		non-specific	182263	122	168	2.36E-06	44.9715	PRK10140	PRK10140	N	cl17182
		**non-specific**	**274661**	**38**	**165**	**3.75E-06**	**44.2719**	**TIGR03585**	**PseH**	**-**	**cl26094**
		**specific**	**173926**	**61**	**126**	**4.81E-05**	**39.1813**	**cd04301**	**NAT_SF**	**-**	**cl17182**
KNH51138	leucyl/phenylalanyl-tRNA-protein transferase	specific	234720	3	237	5.74E-130	366.062	PRK00301	aat	-	cl17182
		**superfamily**	**327402**	**3**	**237**	**5.74E-130**	**366.062**	**cl17182**	**NAT_SF superfamily**	**-**	**-**
		specific	225235	13	232	2.84E-125	353.92	COG2360	Aat	-	cl17182
		specific	273207	34	214	1.37E-114	325.657	TIGR00667	aat	-	cl17182
		specific	308920	37	206	1.23E-100	289.663	pfam03588	Leu_Phe_trans	-	cl17182
KNH49567	ElaA protein	**specific**	**225064**	**1**	**149**	**1.40E-86**	**249.555**	**COG2153**	**ElaA**	**-**	**cl28579**
		**superfamily**	**225064**	**1**	**149**	**1.40E-86**	**249.555**	**cl28579**	**ElaA superfamily**	**-**	**-**
		non-specific	182373	1	148	2.54E-52	162.705	PRK10314	PRK10314	-	cl17182
		**superfamily**	**327402**	**1**	**148**	**2.54E-52**	**162.705**	**cl17182**	**NAT_SF superfamily**	**-**	**-**
		**specific**	**316217**	**38**	**148**	**6.84E-20**	**79.2326**	**pfam13673**	**Acetyltransf_10**	**-**	**cl26092**
		**superfamily**	**330913**	**38**	**148**	**6.84E-20**	**79.2326**	**cl26092**	**Acetyltransf_10 superfamily**	**-**	**-**
		**specific**	**173926**	**50**	**106**	**1.22E-07**	**45.7297**	**cd04301**	**NAT_SF**	**-**	**cl17182**
KNH51202	GNAT family Acetyltransferase	**non-specific**	**236715**	**3**	**122**	**1.81E-18**	**75.8743**	**PRK10562**	**PRK10562**	**-**	**cl17182**
		**superfamily**	**327402**	**3**	**122**	**1.81E-18**	**75.8743**	**cl17182**	**NAT_SF superfamily**	**-**	**-**
		**specific**	**306954**	**11**	**119**	**1.43E-15**	**67.535**	**pfam00583**	**Acetyltransf_1**	**-**	**cl17182**
		**non-specific**	**273701**	**55**	**133**	**2.09E-09**	**51.9459**	**TIGR01575**	**rimI**	**N**	**cl26092**
		**superfamily**	**330913**	**55**	**133**	**2.09E-09**	**51.9459**	**cl26092**	**Acetyltransf_10 superfamily**	**N**	**-**
		specific	223532	1	130	6.30E-09	51.5358	COG0456	RimI	-	cl26092
		**specific**	**173926**	**48**	**92**	**1.39E-07**	**45.3445**	**cd04301**	**NAT_SF**	**C**	**cl17182**
KNH51471	hemolysin	specific	153248	52	261	4.67E-101	304.555	cd07986	LPLAT_ACT14924-like	-	cl17185
		superfamily	327403	52	261	4.67E-101	304.555	cl17185	LPLAT superfamily	-	-
		specific	225717	1	286	1.47E-100	306.247	COG3176	COG3176	-	cl17185
		**specific**	**316007**	**328**	**429**	**3.32E-28**	**108.09**	**pfam13444**	**Acetyltransf_5**	**-**	**cl17185**

		superfamily	327403	328	429	3.32E-28	108.09	cl17185	LPLAT superfamily	-	-
		specific	214724	75	197	1.69E-15	72.773	smart00563	PlsC	-	cl17185
		non-specific	225717	275	574	8.05E-06	47.7779	COG3176	COG3176	-	cl17185
		non-specific	279841	67	158	8.46E-06	45.3471	pfam01553	Acyltransferase	C	cl17185
KNH50323	GNAT family Acetyltransferase	non-specific	211834	481	821	0	598.326	TIGR03536	DapD_gpp	-	cl25962
		superfamily	330783	481	821	0	598.326	cl25962	THDPS_M superfamily	-	-
		specific	314647	157	359	1.94E-107	327.58	pfam11814	DUF3335	-	cl00296
		superfamily	320884	157	359	1.94E-107	327.58	cl00296	Peptidase_C39_like superfamily	-	-
		specific	225082	563	820	3.62E-102	316.555	COG2171	DapD	-	cl25958
		superfamily	330779	563	820	3.62E-102	316.555	cl25958	THDPS_N_2 superfamily	-	-
		specific	100054	658	804	1.88E-83	262.734	cd04649	LbH_THP_succinylT_putative	-	cl00160
		superfamily	320789	658	804	1.88E-83	262.734	cl00160	LbetaH superfamily	-	-
		specific	223532	1	151	4.41E-28	111.242	COG0456	RimI	-	cl26092
		**superfamily**	**330913**	**1**	**151**	**4.41E-28**	**111.242**	**cl26092**	**Acetyltransf_10 superfamily**	**-**	**-**
		**non-specific**	**273701**	**10**	**135**	**2.69E-23**	**95.8586**	**TIGR01575**	**rimI**	**-**	**cl26092**
		**specific**	**306954**	**26**	**123**	**3.36E-18**	**80.6318**	**pfam00583**	**Acetyltransf_1**	**-**	**cl17182**
		**superfamily**	**327402**	**26**	**123**	**3.36E-18**	**80.6318**	**cl17182**	**NAT_SF superfamily**	**-**	**-**
		specific	317226	609	649	3.53E-17	75.2307	pfam14789	THDPS_M	-	cl25962
		non-specific	235142	1	123	2.81E-15	73.039	PRK03624	PRK03624	-	cl26092
		**specific**	**173926**	**43**	**106**	**3.08E-09**	**53.4336**	**cd04301**	**NAT_SF**	**-**	**cl17182**
		non-specific	234858	661	758	2.44E-06	50.1375	PRK00892	lpxD	NC	cl28358
		superfamily	333178	661	758	2.44E-06	50.1375	cl28358	LpxD superfamily	NC	-
KNH51243	hypothetical protein	**specific**	**316217**	**36**	**154**	**1.70E-20**	**83.0846**	**pfam13673**	**Acetyltransf_10**	**-**	**cl26092**
		**superfamily**	**330913**	**36**	**154**	**1.70E-20**	**83.0846**	**cl26092**	**Acetyltransf_10 superfamily**	**-**	**-**
		specific	224584	3	138	6.21E-15	69.977	COG1670	RimL	-	cl26094
		**superfamily**	**330915**	**3**	**138**	**6.21E-15**	**69.977**	**cl26094**	**Acetyltransf_3 superfamily**	**-**	**-**
		**superfamily**	**327402**	**14**	**132**	**8.78E-13**	**62.5274**	**cl17182**	**NAT_SF superfamily**	**-**	**-**
		**non-specific**	**273701**	**31**	**135**	**8.65E-08**	**49.2495**	**TIGR01575**	**rimI**	**-**	**cl26092**
		**specific**	**173926**	**53**	**104**	**0.000359**	**37.6405**	**cd04301**	**NAT_SF**	**-**	**cl17182**

		non-specific	181904	85	137	0.00301	36.831	PRK09491	rimI	N	cl17182
KNH49551	acetyltransferase	**specific**	**315878**	**7**	**139**	**7.90E-16**	**70.0662**	**pfam13302**	**Acetyltransf_3**	**-**	**cl26094**
		**superfamily**	**330915**	**7**	**139**	**7.90E-16**	**70.0662**	**cl26094**	**Acetyltransf_3 superfamily**	**-**	**-**
		specific	224584	1	147	8.48E-15	68.4362	COG1670	RimL	-	cl26094
KNH52093	acetyltransferase gnat family protein	specific	224584	42	139	1.04E-10	56.8802	COG1670	RimL	N	cl26094
		**superfamily**	**330915**	**42**	**139**	**1.04E-10**	**56.8802**	**cl26094**	**Acetyltransf_3 superfamily**	**N**	**-**
		**specific**	**315878**	**64**	**141**	**4.45E-10**	**54.273**	**pfam13302**	**Acetyltransf_3**	**N**	**cl26094**
		non-specific	319965	67	150	0.000412	37.761	cd16358	GlxI_Ni	N	cl14632
		superfamily	326334	67	150	0.000412	37.761	cl14632	VOC superfamily	N	-
KNH49015	GNAT family Acetyltransferase	**specific**	**306954**	**8**	**120**	**1.60E-09**	**52.127**	**pfam00583**	**Acetyltransf_1**	**-**	**cl17182**
		**superfamily**	**327402**	**8**	**120**	**1.60E-09**	**52.127**	**cl17182**	**NAT_SF superfamily**	**-**	**-**
		**specific**	**173926**	**41**	**100**	**1.20E-07**	**45.7297**	**cd04301**	**NAT_SF**	**-**	**cl17182**
		non-specific	131459	30	96	3.35E-06	43.9405	TIGR02406	ectoine_EctA	C	cl17182
		non-specific	182877	70	123	6.58E-06	43.3795	PRK10975	PRK10975	N	cl17182
		**non-specific**	**225910**	**40**	**101**	**2.32E-05**	**42.1125**	**COG3375**	**COG3375**	**C**	**cl17182**
KNH50816	GGDEF family protein	non-specific	131459	784	935	1.78E-61	205.339	TIGR02406	ectoine_EctA	-	cl17182
		**superfamily**	**327402**	**784**	**935**	**1.78E-61**	**205.339**	**cl17182**	**NAT_SF superfamily**	**-**	**-**
		specific	225109	489	643	3.94E-22	94.4395	COG2199	GGDEF	-	cl11967
		superfamily	325147	489	643	3.94E-22	94.4395	cl11967	Nucleotidyl_cyc_III superfamily	-	-
		specific	143635	491	648	8.50E-19	84.1452	cd01949	GGDEF	-	cl11967
		non-specific	128563	489	641	1.76E-16	77.6729	smart00267	GGDEF	-	cl11967
		non-specific	272984	489	647	7.79E-15	72.7549	TIGR00254	GGDEF	-	cl11967
		non-specific	307227	493	645	2.94E-13	68.0422	pfam00990	GGDEF	-	cl11967
		**specific**	**306954**	**823**	**903**	**1.19E-12**	**65.2238**	**pfam00583**	**Acetyltransf_1**	**N**	**cl17182**
		non-specific	274350	200	471	5.24E-12	69.7272	TIGR02917	PEP_TPR_lipo	NC	cl26002
		superfamily	330823	200	471	5.24E-12	69.7272	cl26002	TPR_11 superfamily	NC	-
		non-specific	176974	200	299	1.40E-10	60.7918	CHL00033	ycf3	N	cl26004
		superfamily	330825	200	299	1.40E-10	60.7918	cl26004	PRK02603 superfamily	N	-
		**specific**	**173926**	**824**	**876**	**3.43E-09**	**53.4336**	**cd04301**	**NAT_SF**	**-**	**cl17182**
		non-specific	182070	493	579	4.57E-09	60.4565	PRK09776	PRK09776	NC	cl25447
		superfamily	330268	493	579	4.57E-09	60.4565	cl25447	COG5001 superfamily	NC	-

		specific	223533	193	408	2.78E-07	52.9261	COG0457	TPR	N	cl26002
		specific	315987	225	289	6.68E-07	47.382	pfam13424	TPR_12	-	cl26181
		superfamily	315987	225	289	6.68E-07	47.382	cl26181	TPR_12 superfamily	-	-
		specific	223532	825	903	2.70E-05	45.3727	COG0456	RimI	N	cl26092
		**superfamily**	**330913**	**825**	**903**	**2.70E-05**	**45.3727**	**cl26092**	**Acetyltransf_10 superfamily**	**N**	**-**
KNH51206	acetyltransferase	specific	224584	16	183	9.02E-16	71.1326	COG1670	RimL	-	cl26094
		**superfamily**	**330915**	**16**	**183**	**9.02E-16**	**71.1326**	**cl26094**	**Acetyltransf_3 superfamily**	**-**	**-**
		**specific**	**306954**	**48**	**158**	**7.07E-13**	**61.757**	**pfam00583**	**Acetyltransf_1**	**-**	**cl17182**
		**superfamily**	**327402**	**48**	**158**	**7.07E-13**	**61.757**	**cl17182**	**NAT_SF superfamily**	**-**	**-**
		**non-specific**	**274661**	**53**	**180**	**7.65E-07**	**46.5831**	**TIGR03585**	**PseH**	**-**	**cl26094**
		non-specific	182263	137	183	2.98E-06	44.9715	PRK10140	PRK10140	N	cl17182
		**specific**	**173926**	**76**	**141**	**3.70E-05**	**39.9517**	**cd04301**	**NAT_SF**	**-**	**cl17182**
KNH51870	hypothetical protein	specific	224361	15	677	0	667.928	COG1444	TmcA	-	cl25451
		superfamily	330272	15	677	0	667.928	cl25451	tRNA_bind_3 superfamily	-	-
		**specific**	**310013**	**179**	**344**	**1.11E-74**	**237.452**	**pfam05127**	**Helicase_RecD**	**-**	**cl21455**
		superfamily	328724	179	344	1.11E-74	237.452	cl21455	P-loop_NTPase superfamily	-	-
		**superfamily**	**327402**	**378**	**540**	**1.28E-21**	**93.8491**	**cl17182**	**NAT_SF superfamily**	**-**	**-**
		**specific**	**173926**	**465**	**496**	**2.46E-05**	**42.2629**	**cd04301**	**NAT_SF**	**N**	**cl17182**
		**non-specific**	**273701**	**468**	**491**	**3.52E-05**	**43.8567**	**TIGR01575**	**rimI**	**NC**	**cl26092**
		**superfamily**	**330913**	**468**	**491**	**3.52E-05**	**43.8567**	**cl26092**	**Acetyltransf_10 superfamily**	**NC**	**-**
		non-specific	181904	471	492	0.000188	41.8386	PRK09491	rimI	NC	cl17182
KNH52084	acetyltransferase	**specific**	**306954**	**14**	**134**	**1.10E-15**	**68.3054**	**pfam00583**	**Acetyltransf_1**	**-**	**cl17182**
		**superfamily**	**327402**	**14**	**134**	**1.10E-15**	**68.3054**	**cl17182**	**NAT_SF superfamily**	**-**	**-**
		**specific**	**173926**	**50**	**116**	**1.40E-10**	**53.4336**	**cd04301**	**NAT_SF**	**-**	**cl17182**
		**superfamily**	**330913**	**32**	**137**	**4.54E-10**	**53.8094**	**cl26092**	**Acetyltransf_10 superfamily**	**-**	**-**
		specific	223532	55	143	5.35E-09	51.921	COG0456	RimI	N	cl26092
		**non-specific**	**273701**	**90**	**143**	**5.13E-05**	**40.3899**	**TIGR01575**	**rimI**	**N**	**cl26092**
		non-specific	181904	92	143	0.005125	34.905	PRK09491	rimI	N	cl17182
OFJ38839	acetyltransferase	non-specific	236715	2	39	3.30E-05	38.1248	PRK10562	PRK10562	N	cl17182
		**superfamily**	**327402**	**2**	**39**	**3.30E-05**	**38.1248**	**cl17182**	**NAT_SF superfamily**	**N**	**-**
OFJ39431	GNAT family N-acetyltransferase	**specific**	**315878**	**9**	**141**	**6.11E-16**	**70.4514**	**pfam13302**	**Acetyltransf_3**	**-**	**cl26094**
		**superfamily**	**330915**	**9**	**141**	**6.11E-16**	**70.4514**	**cl26094**	**Acetyltransf_3 superfamily**	**-**	**-**

		specific	224584	1	149	4.72E-15	69.2066	COG1670	RimL	-	cl26094
OFJ40229	acetyltransferase	non-specific	236715	2	39	3.30E-05	38.1248	PRK10562	PRK10562	N	cl17182
		**superfamily**	**327402**	**2**	**39**	**3.30E-05**	**38.1248**	**cl17182**	**NAT_SF superfamily**	**N**	**-**
