# Supplementary figures and images for: The Global Acetylome of the Human Pathogen Vibrio cholerae V52 Reveals Lysine Acetylation of Major Transcriptional Regulators
Source: Front Cell Infect Microbiol. 2018 Jan 11;7:537. doi: 10.3389/fcimb.2017.00537 (PMC5768985; doi:10.3389/fcimb.2017.00537)

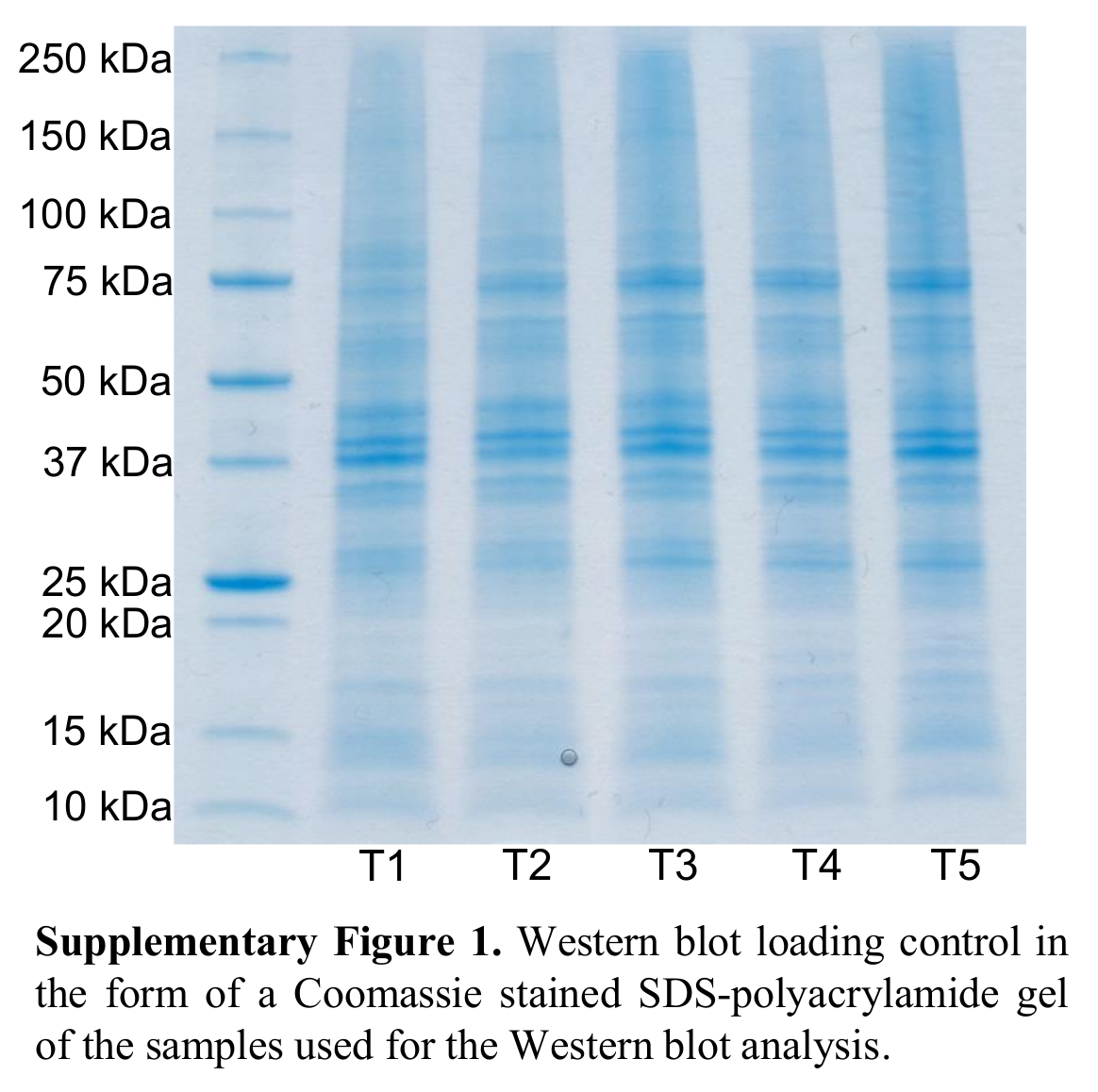

Supplement: Supplementary file 5 [file Image1.TIF]

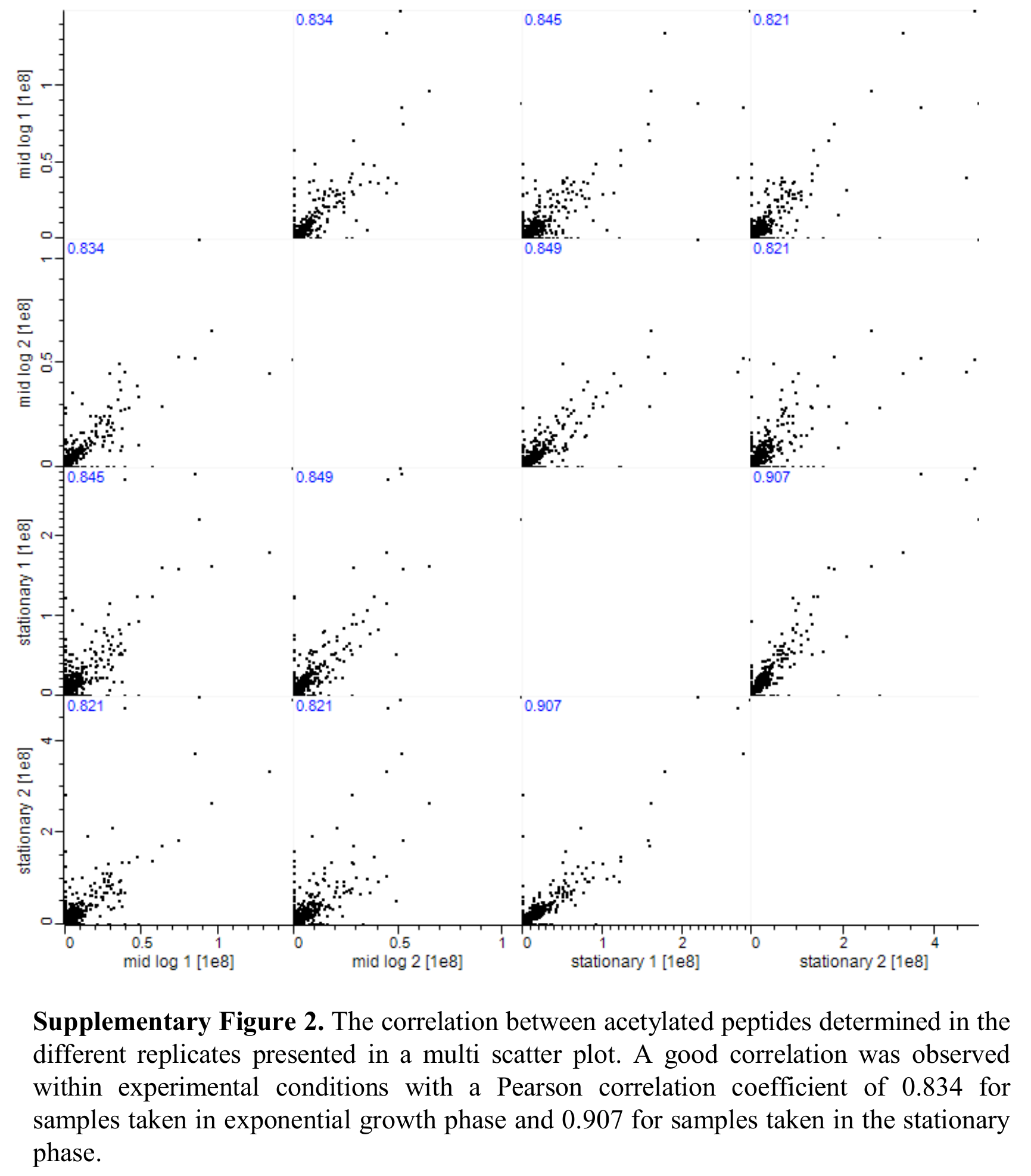

Supplement: Supplementary file 6 [file Image2.TIF]

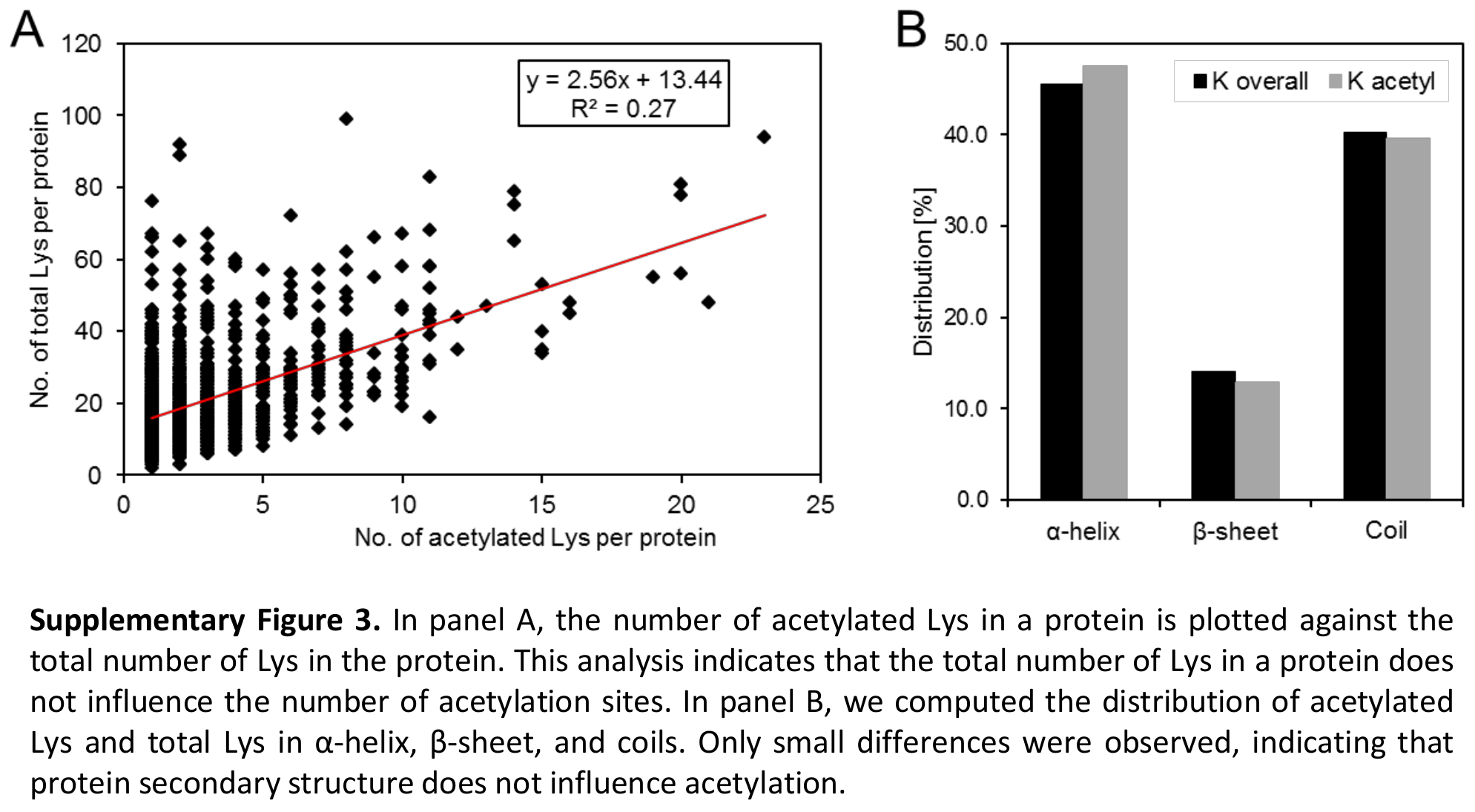

Supplement: Supplementary file 7 [file Image3.TIF]
