# Supplementary material for: The Global Acetylome of the Human Pathogen Vibrio cholerae V52 Reveals Lysine Acetylation of Major Transcriptional Regulators
Source: Front Cell Infect Microbiol. 2018 Jan 11;7:537. doi: 10.3389/fcimb.2017.00537 (PMC5768985; doi:10.3389/fcimb.2017.00537)
Supplement: Supplementary file 8 [file Image4.PDF]

Panel A

```
acetoacetyl-CoA         ------------------MSSTTPLWTP---SHERISAS---NLQQFIAHVNMQGEAIDS    36
B. subtilis             ------------------------------------------------------------    0
Homo sapiens            MAARTLGRGVGRLLGSLRGLSGQPARPPCGVSAPRRAASGPSGSAPAVAA---AAAQPGS    57
acetyl-coA synth        -------------------------------MSEA----HIYPVKQNIKAH---THADNDT    23
E. coli                 -------------------------------MSQI----HKHTIPANIADR---CLINPQQ    23
S. typhimurium          -------------------------------MSQT----HKHAIPANIADR---CLINPEQ    23


acetoacetyl-CoA         YSSLHQWSVAETRQFWLEVWQ-FCDV---IGYRGNCIIGEGLPRFGKTMVPARDSIWFPQ    92
B. subtilis             ---MN---LKALPAIEGDHN--LKNYE---ETYRHFDWAE----------AEKHFSWHET    39
Homo sapiens            YPALSAQAAREPAAFWGPLARDTLVWDTPYHTVWDCDFST----------GK--IGWFLG    105
acetyl-coA synth        YLAMYQQSIKDPEGFWSEHG-KIVDWIKPFTKVKHTSFDP----------GHIDIRWFED    72
E. coli                 YEAMYQQSINVPDTFWGEQG-KILDWIKPYQKVKNTSFAP----------GNVSIKWYED    72
S. typhimurium          YETKYKQSINDPDTFWGEQG-KILDWITPYQKVKNTSFAP----------GNVSIKWYED    72
                                      :                   .                     *.

acetoacetyl-CoA         AQLNYAENLLSYAFQN--PDGIALWFKNENGHTKKFSWQQLCDHVSVVQQWLAQNG-VGE    149
B. subtilis             GKLNAAYEAIDRHAESFRKNKVALYYK-DAKRDEKYTFKEMKEESNRAGNVLRRYGNVEK    98
Homo sapiens            GQLNVSVNCLDQHVRKSPESVALIWERDEPGTEVRITYRELLETTCRLANTLKRHG-VHR    164
acetyl-coA synth        GTLNVSANCIDRHLATRGDQVAIIWEGDDPTQDKTLTYKQLHQEVCRFANALKEQG-VRK    131
E. coli                 GTLNLAANCLDRHLQENGDRTAIIWEGDDASQSKHISYKELHRDVCRFANTLLELG-IKK    131
S. typhimurium          GTLNLAANCLDRHLQENGDRTAIIWEGDDTSQSKHISYRELHRDVCRFANTLLDLG-IKK    131
                        . ** : : :.             ::  :       ::::         : *   * : .

acetoacetyl-CoA         GDVVAGYLPHLPETVIAMLAATSLGAIWTSTSPDFGVESVIERFGQVQPKILFCCNGYTF    209
B. subtilis             GDRVFIFMPRSPELYFIMLGAIKIGAIAGPLFEAFMEGAVKDRLENSEAKVVVTTPELLE    158
Homo sapiens            GDRVAIYMPVSPLAVAAMLACARIGAVHTVIFAGFSAESLAGRINDAKCKVVITFNQGLR    224
acetyl-coA synth        GDVVCIYMPMVPEAAVAMLACTRIGAVHTIVFGGFSPEALAGRIIDSNAKLVITADEGVR    191
E. coli                 GDVVAIYMPMVPEAAVAMLACARIGAVHSVIFGGFSPEAVAGRIIDSNSRLVITSDEGVR    191
S. typhimurium          GDVVAIYMPMVPEAAVAMLACARIGAVHSVIFGGFSPEAVAGRIIDSSSRLVITADEGVR    191
                        ** *  ::*  *     **..  :**:       *    ::  *: : . :::.

acetoacetyl-CoA         NGKSFPMQERNAQIASALPSLVNTCQIEYLQDRNFTPDFNDSFSDWQSIFASYQPRGVEY    269
B. subtilis             RI--------------PVDKLPHLQHVFVVGG---EAESGT-NIINYDEAAKQESTRLDI    200
Homo sapiens            GGRVVELKKIVDE-AVKHC--PTVQHVLVAHRTDNKVHMGD-LDVPLEQEMAKEDPVCAP    280
acetyl-coA synth        GGRAVPLKKNVDE-ALCNPEVKNISKVMVLKRTGGNVAWNEHRDIWWHEATAKASDHCQP    250
E. coli                 AGRSIPLKKNVDD-ALKNPNVTSVEHVVVLKRTGGKIDWQEGRDLWWHDLVEQASDQHQA    250
S. typhimurium          AGRSIPLKKNVDD-ALKNPNVTSVEHVIVLKRTGSDIDWQEGRDLWWRDLIEKASPEHQP    250
                                                 ::

acetoacetyl-CoA         RRIGFNDPLFVLYSSGTTGKPKCITHSVGGTLLNHLKEHQLHCDIQPQDRVFYYTTTGWM    329
B. subtilis             EWMDKKDGFLLHYTSGSTGTPKGVLHVHEAMI-QQYQTGKWVLDLKEEDIYWCTADPGWV    259
Homo sapiens            ESMGSEDMLFMLYTSGSTGMPKGIVHTQAGYLLYAALTHKLVFDHQPGDIFGCVADIGWI    340
acetyl-coA synth        EEMKAEDPLFILYTSGSTGKPKGVLHTTGGYLVYATMTFKYVFDYQPNEVFWCTADVGWI    310
E. coli                 EEMNAEDPLFILYTSGSTGKPKGVLHTTGGYLVYAALTFKYVFDYHPGDIYWCTADVGWV    310
S. typhimurium          EAMNAEDPLFILYTSGSTGKPKGVLHTTGGYLVYAATTFKYVFDYHPGDIYWCTADVGWV    310
                        . :  :* ::: *:**:** ** : *   . :       :   * :  :     :  **:

acetoacetyl-CoA         MWNW--HVSALASGATLVIYDGHPLYPQAGALWALVDEAKVSLFGTSAKYLETLQKNQFS    387
B. subtilis             TGTVYGIFAPWLNGATNVIVGGRF---SPESWYGTIEQLGVNVWYSAPTAFRMLMGAGDE    316
Homo sapiens            TGHSYVVYGPLCNGATSVLFESTPVYPNAGRYWETVERLKINQFYGAPTAVRLLLKYGDA    400
acetyl-coA synth        TGHSYLVYGPLANGAKTILFEGVPNYPTTARMSEVVDKHKVNILYTAPTAIRALMAKGDE    370
E. coli                 TGHSYLLYGPLACGATTLMFEGVPNWPTPARMAQVVDKHQVNILYTAPTAIRALMAEGDK    370
S. typhimurium          TGHSYLLYGPLACGATTLMFEGVPNWPTPARMCQVVDKHQVNILYTAPTAIRALMAEGDK    370
                                .    **. ::  .             ::.  :.    : . .. *

acetoacetyl-CoA         PCDFYSLSHLKTLCSTGSVLYPEQFDYVYEHVKSDL------HLASISGGTDICGCFVLG    441
B. subtilis             MAAKYDLTSLRHVLSVGEPLNPEVIRWGHKVFNK---RIHDTWWMTETGSQLICNY--PC    371
Homo sapiens            WVKKYDRSSLRTLGSVGEPINCEAWEWLHRVVGDSRCTLVDTWWQTETGGICIAPR--PS    458
acetyl-coA synth        AIKGTSRDSLRIMGSVGEPINPEAWEWYYRTIGNEKSPIVDTWWQTETGGILITPL--PG    428
E. coli                 AIEGTDRSSLRILGSVGEPINPEAWEWYWKKIGNEKCPVVDTWWQTETGGFMITPL--PG    428
S. typhimurium          AIEGTDRSSLRILGSVGEPINPEAWEWYWKKIGKEKCPVVDTWWQTETGGFMITPL--PG    428
```

```
                       .   *: : *.*. :  *   :  . . .          : :*.  *

acetoacetyl-CoA      NPISPVYQGECQSAGLGLDVVAYNQHGEAIVA--ERGELVCRNSFPNQPIGFWHDDGSRY   499
B. subtilis          M---DIKPGSMGKPIPGVEAAIVDNQGNELPPYR-MGNLAIKKGWPSMMHTIWNN-PEKY   426
Homo sapiens         EEGAEILPAMAMRPFFGIVPVLMDEKGSVVEGSNVSGALCISQAWPGMARTIYGD-HQRF   517
acetyl-coA synth     A--TALKPGSATRPFFGVQPALVDNMGEIVEGAT-EGNLVLLDSWPGQMRTVYGD-HDRF   484
E. coli              A--TELKAGSATRPFFGVQPALVDNEGNPLEGAT-EGSLVITDSWPGQARTLFGD-HERF   484
S. typhimurium       A--IELKAGSATRPFFGVQPALVDNEGHPQEGAT-EGNLVITDSWPGQARTLFGD-HERF   484
                         :  .       *:  .  :: *         * *   ..:*.    .: :  .::

acetoacetyl-CoA      HQAYWDKYPGVWHHGDEIEITDKGGVLFFGRSDTVLNPGGVRIGTAEIYQQVNALPEIHD   559
B. subtilis          ESYFM--PGGWYVSGDSAYMDEEGYFWFQGRVDDVIMTSGERVGPFEVESKLVEHPAIAE   484
Homo sapiens         VDAYFKAYPGYYFTGDGAYRTEGGYYQITGRMDDVINISGHRLGTAEIEDAIADHPAVPE   577
acetyl-coA synth     EQTYFSTFKGMYFTGDGARRDEDGYYWITGRVDDVLNVSGHRMGTAEIESALVAFNKIAE   544
E. coli              EQTYFSTFKNMYFSGDGARRDEDGYYWITGRVDDVLNVSGHRLGTAEIESALVAHPKIAE   544
S. typhimurium       EQTYFSTFKNMYFSGDGARRDEDGYYWITGRVDDVLNVSGHRLGTAEIESALVAHPKIAE   544
                      . :     . :  **     : *   : ** * *:  .* *:*   *: . :     : :

acetoacetyl-CoA      SIAIGRHIDR-DEQVILFVQLAQNVPFNDELQQKIRSLLRERCSPRHVPAHIYAISEIPR   618
B. subtilis          AGVIGKPDPVRGEIIKAFIALREGFEPSDKLKEEIRLFVKQGLAAHAAPREIEFKDKLPK   544
Homo sapiens         SAVIGYPHDIKGEAAFAFIVVKDSAGDSDVVVQELKSMVATKIAKYAVPDEILVVKRLPK   637
acetyl-coA synth     AAVVGVPHDIKGQAIYAYITLNDGVYPSAELHKEVKDWVRKEIGAIATPDVLHWTDALPK   604
E. coli              AAVVGIPHNIKGQAIYAYVTLNHGEEPSPELYAEVRNWVRKEIGPLATPDVLHWTDSLPK   604
S. typhimurium       AAVVGIPHAIKGQAIYAYVTLNHGEEPSPELYAEVRNWVRKEIGPLATPDVLHWTDSLPK   604
                     : .:*      .:    :: : ..   .  :  :::  :     .    .*  :   . :*:

acetoacetyl-CoA      TKSGKLVELAVKQVCHGDE--VKNLGAIANPQVLAEIERLLTA---------   659
B. subtilis          TRSGKIMRRVLKAWELN--LPAGDLSTMED---------------------   572
Homo sapiens         TRSGKVMRRLLRKIITSEAQELGDTTTLEDPSIIAEILSVYQKCKDKQAAAK   689
acetyl-coA synth     TRSGKIMRRILRKIATGDTSNLGDTSTLADPSVVDRLIAEKAQLK-------   649
E. coli              TRSGKIMRRILRKIAAGDTSNLGDTSTLADPGVVEKLLEEKQAIAMPS----   652
S. typhimurium       TRSGKIMRRILRKIAAGDTSNLGDTSTLADPGVVEKLLEEKQAIAMPS----   652
                     *:***::.  ::    .      :  :: :
```

Panel B

```
Malate DH        1  MKVAVIGAAGGIGQALALLLKNRLPAGSDLALYDIAPVTPGVAADLSHIP    50
                    |||||:||||||||||||||||.:||:||:|:|||||||||||||.||||||
E. coli          1  MKVAVLGAAGGIGQALALLLKTQLPSGSELSLYDIAPVTPGVAVDLSHIP    50

Malate DH       51  TPVTIKGYAGEDPTPALEGADVVLVSAGVARKPGMDRADLFNVNAGIVKA   100
                    |.|.|||::|||.||||||||||||:||||||||||||:|||||||||||.
E. coli         51  TAVKIKGFSGEDATPALEGADVVLISAGVARKPGMDRSDLFNVNAGIVKN   100

Malate DH      101  LAEKIAVVCPKACVGIITNPVNTTVPIAAEVLKKAGVYDKRKLFGVTTLD   150
                    |.:::|..|||||:||||||||||||.||||||||||||||.|||||||||
E. coli        101  LVQQVAKTCPKACIGIITNPVNTTVAIAAEVLKKAGVYDKNKLFGVTTLD   150

Malate DH      151  VIRSETFVAALKDKDPGQVRVPVIGGHSGVTILPLLSQVEGVSFTDEEVA   200
                    :|||.||||.||.|.||:|.||||||||||||||||||.|||||::|||
E. coli        151  IIRSNTFVAELKGKQPGEVEVPVIGGHSGVTILPLLSQVPGVSFTEQEVA   200

Malate DH      201  ALTKRIQNAGTEVVEAKAGGGSATLSMGQAACRFGLALVKALQGESDVVE   250
                    .|||||||||||||||||||||||||||||||.|||||:||:|||||..|||
E. coli        201  DLTKRIQNAGTEVVEAKAGGGSATLSMGQAAARFGLSLVRALQGEQGVVE   250

Malate DH      251  YAYVEGEGEYAPFFAQPIKLGKNGVEALLDIGKLSAYEQAALDGMLDTLK   300
                    .|||||:|:|||.||:||:.||||||||....|||.||||:|||.||:|||||||
E. coli        251  CAYVEGDGQYARFFSQPLLLGKNGVEERKSIGTLSAFEQNALEGMLDTLK   300

Malate DH      301  GDIQIGVEFV-K    311
                    .||.:|.|||  |
E. coli        301  KDIALGEEFVNK    312
```

Panel C

```
SAM Synthase       1 MAIKHLFTSESVSEGHPDKIADQISDAVLDAIFEQDPKARVACETYVKTG   50
                     || |||||||||||||||||||||||||||||.|||||||||||||||||
E. coli            1 MA-KHLFTSESVSEGHPDKIADQISDAVLDAILEQDPKARVACETYVKTG   49

SAM SYNTHASE      51 MVMVGGEITTSAWVDIEEITRQTVREIGYVHSDMGFDANSCAVLNTIGKQ  100
                     ||:||||||||||||||||||.||||||||||||||||||||||:.||||
E. coli           50 MVLVGGEITTSAWVDIEEITRNTVREIGYVHSDMGFDANSCAVLSAIGKQ   99

SAM SYNTHASE     101 SPDINQGVDKADPKEQGAGDQGIMFGYATNETEVLMPAPITYAHRLMQRQ  150
                     |||||||||:|||.||||||||:|||||||||:|||||||||||||:|||
E. coli          100 SPDINQGVDRADPLEQGAGDQGLMFGYATNETDVLMPAPITYAHRLVQRQ  149

SAM SYNTHASE     151 AEVRKNGTLPWLRPDAKSQVTFQYDQGKIVGIDAVVLSTQHSDSISTADL  200
                     |||||||||||||||||||||||||.||||||||||||||||:.|....|
E. coli          150 AEVRKNGTLPWLRPDAKSQVTFQYDDGKIVGIDAVVLSTQHSEEIDQKSL  199

SAM SYNTHASE       1 REAVMEEIIKPVLPAEWLSKETKYFINPTGRFVIGGPMGDCGLTGRKIIV  250
                     :||||||||||:||||||:..||:||||||||||||||||||||||||||
E. coli          200 QEAVMEEIIKPILPAEWLTSATKFFINPTGRFVIGGPMGDCGLTGRKIIV  249

SAM SYNTHASE     251 DTYGGAARHGGGAFSGKDPSKVDRSAAYAARYVAKNIVAAGMADRCEIQL  300
                     |||||.|||||||||||||||||||||||||||||||||||:|||||||:
E. coli          250 DTYGGMARHGGGAFSGKDPSKVDRSAAYAARYVAKNIVAAGLADRCEIQV  299

SAM SYNTHASE     301 SYAIGVADPTSIMVETFGTEKVSQEIIIEAVRQFFDLRPYGLQEMLNLLQ  350
                     |||||||:||||||||||||||..|.:...||:|||||||||.:||:||.
E. coli          300 SYAIGVAEPTSIMVETFGTEKVPSEQLTLLVREFFDLRPYGLIQMLDLLH  349

SAM SYNTHASE     351 PIYKKTAAYGHFGREEFPWEATDKAALLRDFAGLK  385
                     ||||:||||||||||.||||.||||.||||.||||
E. coli          350 PIYKETAAYGHFGREHFPWEKTDKAQLLRDAAGLK  384
```

Panel D

```
USP                1 MYKHILVPVDLNEQGFADKAVQLAVWHAKHSNAEIHLLNVLPGIHMSMVA   50
                          ::.|..:...|...||:..:..||..:..:.::|...|  .:.|.
M. smegmatis       1 -----MIVVGYSADPFGRAAVEHGIEEAKRRDTGLLVINATAG--DAYVD   43

USP               51 TYFPKDAAAQMKNDVRAQLKAFAEKHIAEEVVYKLHIAEGKPYATILDYA  100
                     ..|.:....   :||.|.|:   :..:..|:...:.:...:...|.:|..
M. smegmatis      44 ARFARSGEV---HDVEAHLQ---DSGVPFEIRQPVGVDATEELLTAMDSP   87

USP              101 ERLGADLIVMPSHKRSRIDKVMLGSVASKVVENSPINVMVVKPQG-  145
                     :   |:|:|:....|:.:.|::|||||.::....|..|:.|||.|
M. smegmatis      88 D---AELLVIGIRHRNPVGKLLLGSVAQRLLLECPKPVLAVKPHGF  130
```

Panel E

```
Leucine RRP        1 MVDSYKKPSKDLDRIDRNILNELQKDGRISNVELSKRVGLSPTPCLERVR   50
                     ||||.|:|.|||||||||||||||||||||||||||||||||||||||||
S. typhimurium     1 MVDSKKRPGKDLDRIDRNILNELQKDGRISNVELSKRVGLSPTPCLERVR   50

Leucine RRP       51 RLERQGFITGYTALLNPQYLDASLLVFVEITLNRGAPDVFEQFNAAVQKL  100
                     ||||||||.||||||||.||||||||||||||||||||||||||||||||
S. typhimurium    51 RLERQGFIQGYTALLNPHYLDASLLVFVEITLNRGAPDVFEQFNAAVQKL  100

Leucine RRP     101 DDIQECHLVSGDFDYLLKTRVSDMGAYRRLLGDTLLRLPGVNDTRTYVVM  150
                     ::|||||||||||||||||||.||.|||:|||:|||||||||||||||||
```

```
S. typhimurium     101  EEIQECHLVSGDFDYLLKTRVPDMSAYRKLLGETLLRLPGVNDTRTYVVM    150

Leucine RRP        151  EEVKQTNQLVIKTR    164
                        |||||:|:||||||
S. typhimurium     151  EEVKQSNRLVIKTR    164
```

Supplementary Figure 4. Alignments of *V. cholerae* proteins with proteins that have been characterized and shown to be regulated by lysine acetylation. Panel A shows the *V. cholerae* acetyl-coenzyme A synthetase (KNH49371) and acetoacetyl-coenzyme A synthetase (KNH51540) aligned with acetyl coenzyme A synthetase from *Bacillus subtilis* (P39062), *Escherichia coli* (P27550), *Salmonella typhimurium* (Q8ZKF6), and human (Q9NUB1). Panel B shows the *V. cholerae* malate dehydrogenase (KNH48740) aligned with that of *Escherichia coli* (P61889). Panel C shows the *V. cholerae* S-adenosylmethionine synthetase (KNH48827) aligned with that of *Escherichia coli* (P0A817). Panel D shows the *V. cholerae* universal stress protein (KNH48736) aligned with that of *Mycobacterium smegmatis* (A0QZZ6). Panel E shows the *V. cholerae* leucine responsive regulatory protein (KNH50497) aligned with that of *Salmonella typhimurium* (P0A2S0). Regulatory sites highlighted with blue, and sites acetylated in *V. cholerae* highlighted in red.
